# Transcriptomic signatures in response to antioxidants supplementation in Korean cattle beef, *Hanwoo*: a 7-month feeding study

**DOI:** 10.3389/fvets.2025.1546248

**Published:** 2025-04-24

**Authors:** Kangwook Lee, La Yoon Choi, Jun Sang Ahn, Jae Yong Song, Joong Kook Park, Suk Jun Yun, Jeong Heon Lee, Eui-Cheol Shin, Soo-Jin Yeom, Jiangchao Zhao, Tae Jin Cho, Nam Su Oh, Jeong-Oh Shin, Dahye Kim, Tae Gyun Kim, Hyung Taek Cho, Hyo Ri Shin, Young Jun Kim, Jae Kyeom Kim

**Affiliations:** ^1^Department of Food and Biotechnology, Korea University, Sejong, Republic of Korea; ^2^Nonghyup Feed, Seoul, Republic of Korea; ^3^Department of GreenBio Science, Gyeongsang National University, Jinju, Republic of Korea; ^4^School of Biological Sciences and Technology, Chonnam National University, Gwangju, Republic of Korea; ^5^Department of Animal Science, Dale Bumpers College of Agricultural, Food and Life Sciences, University of Arkansas, Fayetteville, AR, United States; ^6^Department of Anatomy, College of Medicine, Soonchunhyang University, Cheonan, Republic of Korea; ^7^Animal Genomics and Bioinformatics Division, National Institute of Animal Science, Rural Development Administration, Wanju, Republic of Korea; ^8^The Bioinformatix, Gwangmyeong, Republic of Korea; ^9^Department of Behavioral Health and Nutrition, University of Delaware, Newark, NJ, United States

**Keywords:** *Hanwoo* cattle, antioxidant supplementation, transcriptomics, RNA sequencing, mitochondrial function

## Abstract

**Introduction:**

The present study investigated the effects of antioxidant supplementation on the transcriptomic profiles of *Hanwoo* cattle during a 7-month feeding trial.

**Methods:**

Twelve castrated *Hanwoo* cattle were randomly assigned to two groups: a control group (CON) and a group supplemented with antioxidants (FEED), consisting of vitamin C, vitamin E, and selenium. Growth performance and carcass traits were evaluated, and liver transcriptomic changes were assessed using RNA sequencing.

**Results and discussion:**

While no significant differences were observed in phenotypic traits such as weight gain and feed conversion ratio, transcriptomic analysis identified 641 differentially expressed genes between the CON and FEED groups. Functional enrichment analysis revealed that differentially expressed genes were mainly associated with transcription regulation, pseudouridine synthesis, and mitochondrial function. These findings suggest that antioxidant supplementation elicits significant molecular changes in the liver, particularly affecting transcriptional activity and mitochondrial processes, even in the absence of detectable phenotypic differences.

## 1 Introduction

The cattle beef industry aims to reduce the fattening period considering several factors such as feed costs and turnover rates of livestock. However, shortening the fattening period has led to a decrease in total animal weight and intramuscular fat (also known as marbling), resulting in reduced farm income. It has been reported that the status of intracellular antioxidants, such as vitamin C, influences the proliferation and differentiation of fat cells and exhibits antioxidant effects by scavenging reactive oxygen species (1). Antioxidants, including vitamin C, play pivotal roles not only in enhancing marbling and beef quality but also in modulating physiological processes at the molecular level. These molecular changes may have broader implications for improving the overall health and productivity of beef cattle. For instance, Pogge et al. (2) found that supplemental vitamin C improved marbling scores in feedlot cattle consuming high sulfur diets. The study suggested that vitamin C might protect protease *μ*-calpain, which is involved in muscle tenderness, and improve the fatty acid profile of the meat by increasing omega-3 and omega-6 fatty acids and decreasing saturated fatty acids (3). Also, it was reported that stress and disease conditions can decrease the levels of ascorbic acid in blood and other tissues while supplementation with vitamin C has been shown to have favorable responses, such as early recovery from stress and disease [reviewed in (4)]. Furthermore, in fattening cattle, as age increases, the endogenous synthesis levels of vitamin C decrease, necessitating supplementation of antioxidants for feeding beef cattle (4).

The transcriptome encompasses the complete set of RNA molecules, including messenger RNA, non-coding RNA, and other functional RNA species, present in a biological sample at a specific point in time. RNA sequencing (RNA-seq) is a powerful molecular biology technique used to analyze and quantify the transcriptome (5). Combined with bioinformatics analyses, transcriptomics can uncover previously unknown pathways, potential druggable targets, and biomarkers involved in tissue-specific responses to various interventions, as we previously reported (6). In this study, we employed RNA-seq as an unbiased and comprehensive method to investigate the effects of supplementation of antioxidants on transcriptomic signatures in the liver of *Hanwoo* cattle. The liver was selected for transcriptomic analysis due to its pivotal role in metabolism, nutrient processing, and stress response, which are directly influenced by antioxidant supplementation. While meat quality is the primary focus in beef production, hepatic transcriptomic profiling offers systemic insights that could indirectly impact muscle development and overall beef quality. In conjunction with the hepatic transcriptomic signature, we have assessed the growth performance and carcass characteristics of Korean fattening cattle according to the feed administered.

## 2 Materials and methods

### 2.1 *Hanwoo* cattle intervention study

The entire study was conducted at the Nonghyup Feed Anseong farm, utilizing a total of 12 castrated *Hanwoo* cattle in the late fattening stage (23 months old). The initial average animal weight was 693.6 ± 61.2 kg. For the feeding trial, the *Hanwoo* cattle were randomly divided into two groups: the control group (CON, *n* = 6) and the antioxidants supplemented group (FEED, *n* = 6). The animals were housed in pens with four cattle per pen, and commercial feeds were provided twice daily (at 8 AM and 4 PM) according to the feed manufacturer’s program, which included mixed feed and roughage. The antioxidant supplementation was top-dressed at 50 g per animal during feedings. Within the 50 g serving, vitamin C made up 99.33%, followed by vitamin E (0.63%) and organic selenium (0.04%). The fixed dosage of 50 g per animal was selected based on supplementation ranges (30–50 g per animal) recommended by the feed manufacturer. This dosage was determined to ensure sufficient antioxidant intake while maintaining safety and consistency across all subjects. Water was freely available. Other management practices followed the farm’s standard protocols. The nutritional composition of the control feeds is presented in Table 1. All the cattle were sacrificed at their age of 30 months.

**Table 1:** Compositions of control feed (contents based on raw materials).

Constituents	Mixed feed	Roughage
Dry matter	10.91	8.23
Crude protein	14.53	9.05
Crude fat	4.10	2.01
Ash meal	6.01	5.19
Crude fiber	5.99	29.82
NDF^1^	29.58	61.25
ADF^2^	11.22	42.15

1 NDF, neutral detergent fiber.

2 ADF, acid detergent fiber.

### 2.2 Growth performance

Body weight was measured monthly before morning feeding using a digital cattle scale, and average daily gain was calculated using the weight gain and number of days reared. Feed intake was calculated monthly for three consecutive days based on the difference between the amount of mixed feed and roughage provided and the leftover, and feed conversion ratio was calculated using dry matter intake and daily gain.

### 2.3 Ultrasound biometric analysis and other characteristics

Ultrasound biometric analyses were conducted at the same sites used for carcass grading (between the last thoracic and first lumbar vertebrae) using ultrasound tomography equipment (set up for 3.5 Hz and180 mm; Honda Electronics, Tokyo, Japan) to evaluate intramuscular fat, ribeye area, and backfat thickness of the animals. After, all animals were shipped to the slaughterhouse at the end of the feeding trial and evaluated by a meat grading professional according to the Livestock Product Grading Standards. Carcass traits were assessed on chilled carcasses 24 h post-slaughter and classified into meat quality and yield grades.

### 2.4 RNA extraction, library construction, and RNA sequencing

To evaluate the transcriptomic changes in *Hanwoo* cattle fed with antioxidants supplements, liver tissues (approximately 50 g per animal) were collected for RNA extraction using the RNeasy mini kit (Qiagen), following the manufacturer’s instructions. DNase I (Qiagen) was used for on-column DNase digestion to eliminate any potential genomic DNA contamination during RNA purification. The quality of the extracted RNA was verified using the RNA Nano 6000 Assay kit and the Bioanalyzer (Agilent Technologies; Santa Clara, CA, USA). For library preparation, the total RNA was fragmented and converted into cDNA. This cDNA underwent end repair, A-tailing, and ligation with indexed adapters. The resulting product was PCR amplified and the cDNA fragments were cleaned up using gel purification to remove any remaining primers and adapters. Transcriptome sequencing was performed on the Illumina NovaSeq platform, generating 150 bp paired-end reads.

### 2.5 Raw data processing

The FASTQ files were first trimmed using the Trimmomatic software. The trimmed FASTQ files were then subjected to FASTQC software to ensure quality of individual raw data is good for further data processing. Afterwards, trimmed FASTQ files were indexed and mapped using the Hisat2. Subsequently, read count was acquired using the FeatureCounts software, followed by differential gene expression analysis using DESeq2 software in R package. DESeq2’s median of ratios was applied to differential gene expression analysis as a scaling normalization method.

### 2.6 Differentially expressed genes (DEG) analysis

The DEG list was produced using the DESeq2 R package, operating under the assumption that there are no differentially expressed genes. The data normalization involved using the median of ratios method, where counts are divided by sample-specific size factors, these being determined by the median ratio of gene counts to the geometric mean per gene. For this study, we aimed to include as many genes as possible for comprehensive bioinformatics analyses thus the default threshold set was *p* < 0.05 without considering their basal gene expression counts and fold changes between groups.

### 2.7 Volcano plot and heatmap analyses

The transcriptomics datasets were first subjected to univariate analyses for exploratory data examination and visualized through volcano plots. Furthermore, to illustrate the relationships between variable sets, multi-dimensional scaling analysis was employed, aiding in the detection of both similarities and differences. Subsequent analyses produced concise mRNA lists, identified as DEGs for *Hanwoo* cattle liver tissues, using the EdgeR tools in R under the premise of no differential gene expression. The EdgeR method adjusts the data by employing the trimmed mean of M-values, calculated as the weighted average of log-ratios between the test and reference samples, excluding the most highly expressed genes and those with the largest log-ratios.

The identified DEGs were also utilized to generate heatmap visualization with hierarchical clustering analysis (HCA). In the HCA, each sample begins as a separate cluster and the algorithm proceeds to combine them until all samples belong to one cluster. Two parameters need to be considered when performing hierarchical clustering. The first one is the similarity measure - Euclidean distance, Pearson’s correlation, and Spearman’s rank correlation. The other parameter is clustering algorithms, including average linkage (clustering uses the centroids of the observations), complete linkage (clustering uses the farthest pair of observations between the two groups), single linkage (clustering uses the closest pair of observations), and Ward’s linkage (clustering to minimize the sum of squares of any two clusters). Heatmap is often presented as a visual aid in addition to the dendrogram. Hierarchical clustering with a heatmap is presented below to visualize the differences between the CON and FEED groups.

### 2.8 Statistical analyses and bioinformatics analyses

Enrichment analyses were conducted on the DEGs from the dataset using Gene Ontology (GO) tools and Kyoto Encyclopedia of Genes and Genomes (KEGG) pathway analysis. Despite using the same DEGs, different databases might highlight various pathways differently based on their specific data and algorithms. The significance of enriched GO terms and KEGG pathways was assessed using adjusted *p*-values (*q*-values) calculated by the Benjamini-Hochberg method, with a false discovery rate (FDR) threshold of <0.05 applied to ensure statistical robustness. Our enrichment analysis concentrated on the three main GO categories: biological processes (GO_BP), cellular components (GO_CC), and molecular functions (GO_MF), and incorporated pathways from the KEGG. Statistical analyses for other phenotype indicators were performed using GraphPad Prism software (Boston, MA, USA). The Shapiro–Wilk test was utilized to determine if the data adhered to a normal distribution. For data that deviated from normality, the Mann–Whitney test was applied. In contrast, data that conformed to a normal distribution were analyzed using a two-tailed unpaired Student’s t-test with Welch’s correction. Data were presented as mean ± standard deviation (SD), and findings were deemed statistically significant when *p*-values were below 0.05.

## 3 Results and discussion

### 3.1 Impacts of antioxidant supplementation on phenotypic characteristics in *Hanwoo* cattle

**Figure 1:**
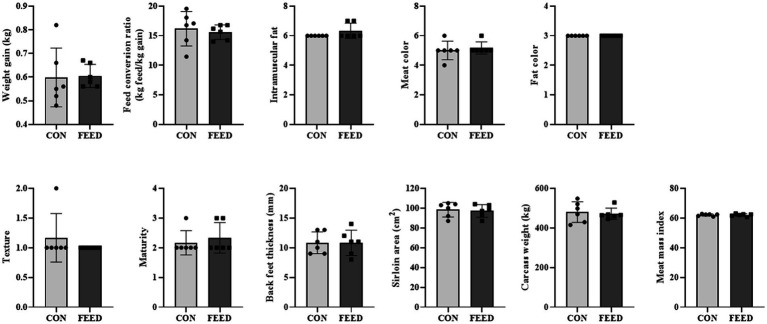
Comparison of phenotypic traits across different dietary intervention groups in *Hanwoo* cattle. Comparative analysis of weight gain, feed conversion ratio, intramuscular fat content, meat color, fat color, texture, maturity, black feet thickness, sirloin area, carcass weight, and meat mass index among cattle subjected to varying dietary interventions. The Shapiro–Wilk test was used to assess normality, and the significance of differences was assessed using an unpaired two-tailed Student’s t-test with Welch’s correction for adjustments. No statistically significant differences were observed across the tested concentrations. Data are presented as means ± standard deviations (*n* = 6). CON, *Hanwoo* cattle fed a standard diet; FEED, *Hanwoo* cattle fed a standard diet supplemented with antioxidants.

First the weight gain per day and feed conversion ratio were assessed. The CON and FEED groups had similar weight gain (0.59 ± 0.12 kg/day vs. 0.61 ± 0.05 kg/day) and feed conversion ratio (16.0 ± 3.25 kg feed/kg gain vs. 15.8 ± 1.14 feed/kg gain) both of which were not statistically different (*p* > 0.05 for both; Figure 1). In addition, other meat quality parameters were assessed including intramuscular fat, meat color, fat color, texture, maturity, backfat thickness, ribeye area, carcass weight, and meat mass index (Figure 1); all the parameters were not different between the CON and FEED groups (*p* > 0.05 for all parameters). The current study observed that the weight gain per day and feed conversion ratio were not significantly different between the control group and the group supplemented with antioxidants (i.e., FEED group). This aligns with findings from similar research. For instance, the effects of vitamin E supplementation on beef cattle were investigated yet there were no significant differences in disease incidence, immune competence, or weight gain when comparing high and normal vitamin E status, suggesting that excess vitamin supplementation may not always yield performance benefits at least on phenotype levels (7).

### 3.2 RNA extraction and sequencing quality control

In order to monitor quality of our sequencing, first we checked the RNA quality where all the samples’ RNA integrity number (also known as RIN) were higher than 7.00 (Supplementary Table S1). RNA concentrations as well as purity results (as assessed via 260/230 and 260/280) were also satisfactory. After, the sequence error rate was calculated using the Phred score where Phred scores 10, 20, 30, and 40 correspond to 10, 1, 0.1, and 0.01% error rates. Following the paired-end transcriptome sequencing analysis of 12 samples, results for all samples fell within the anticipated range. The Supplementary Table S2 shows the raw and processed reads for each sample, categorized by total data volume and Q30 (Phred score for base quality), demonstrating metrics that exceed a value of 30 (Supplementary Table S2).

### 3.3 DEG establishment, volcano plot, and heatmap analyses

**Figure 2:**
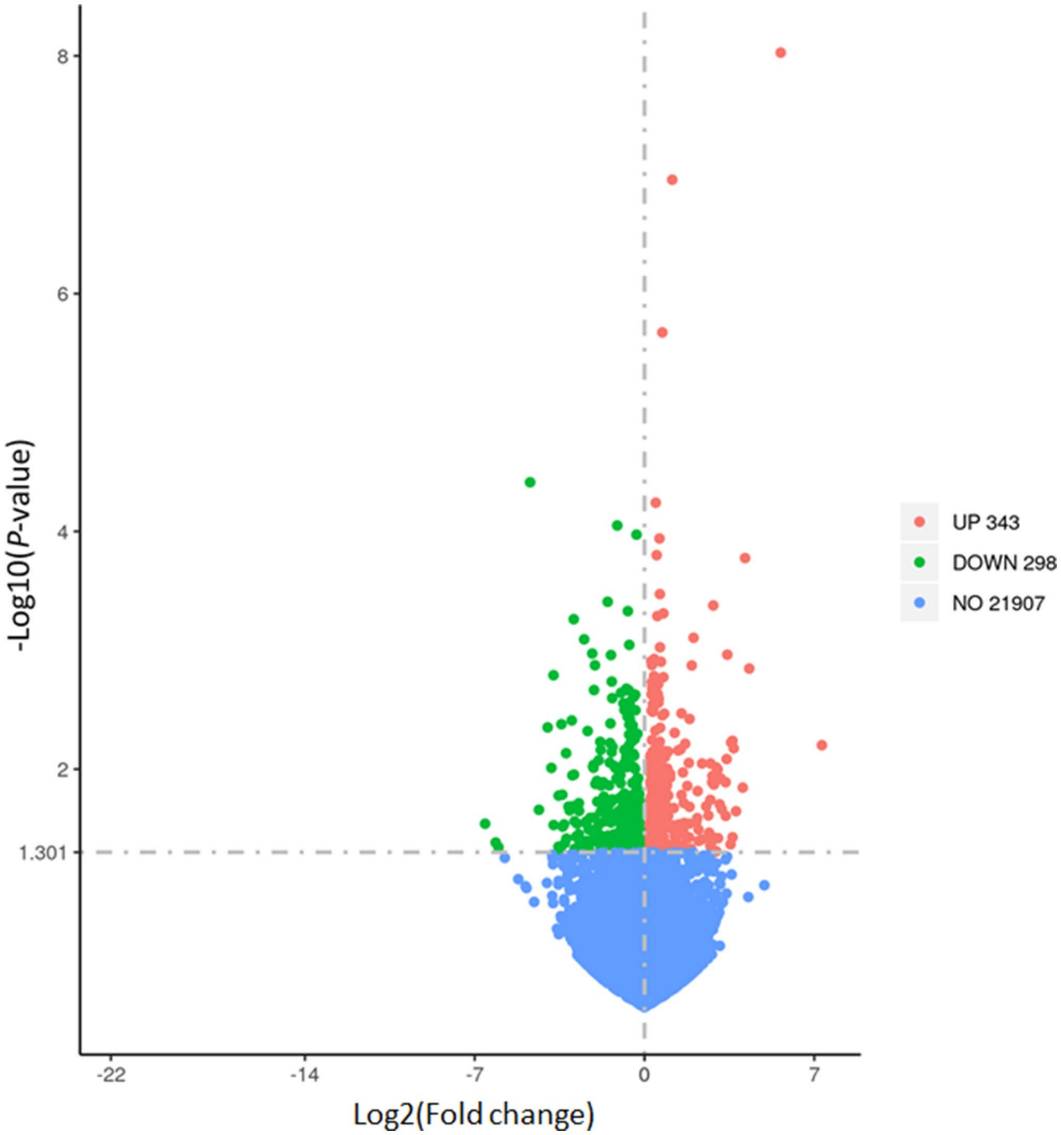
Volcano plot of differential gene expression (DEG) in liver tissues of *Hanwoo* cattle fed with different dietary intervention. The vertical axis (*y*-axis) corresponds to the –log_10_ (False discovery rate), and the horizontal axis (*x*-axis) displays the log_2_ fold change (logFC) value. Each point represents an individual gene, plotted by log2 fold change on the *x*-axis and −log10 *p*-value on the *y*-axis. Genes significantly upregulated in the treatment group compared to the control group are depicted in red on the right, while those significantly downregulated are shown in green on the left. The horizontal dashed line indicates the threshold for statistical significance (*p* < 0.05), and the vertical dashed lines mark the cut-off for log2 fold change. DEG, differential gene expression.

**Figure 3:**
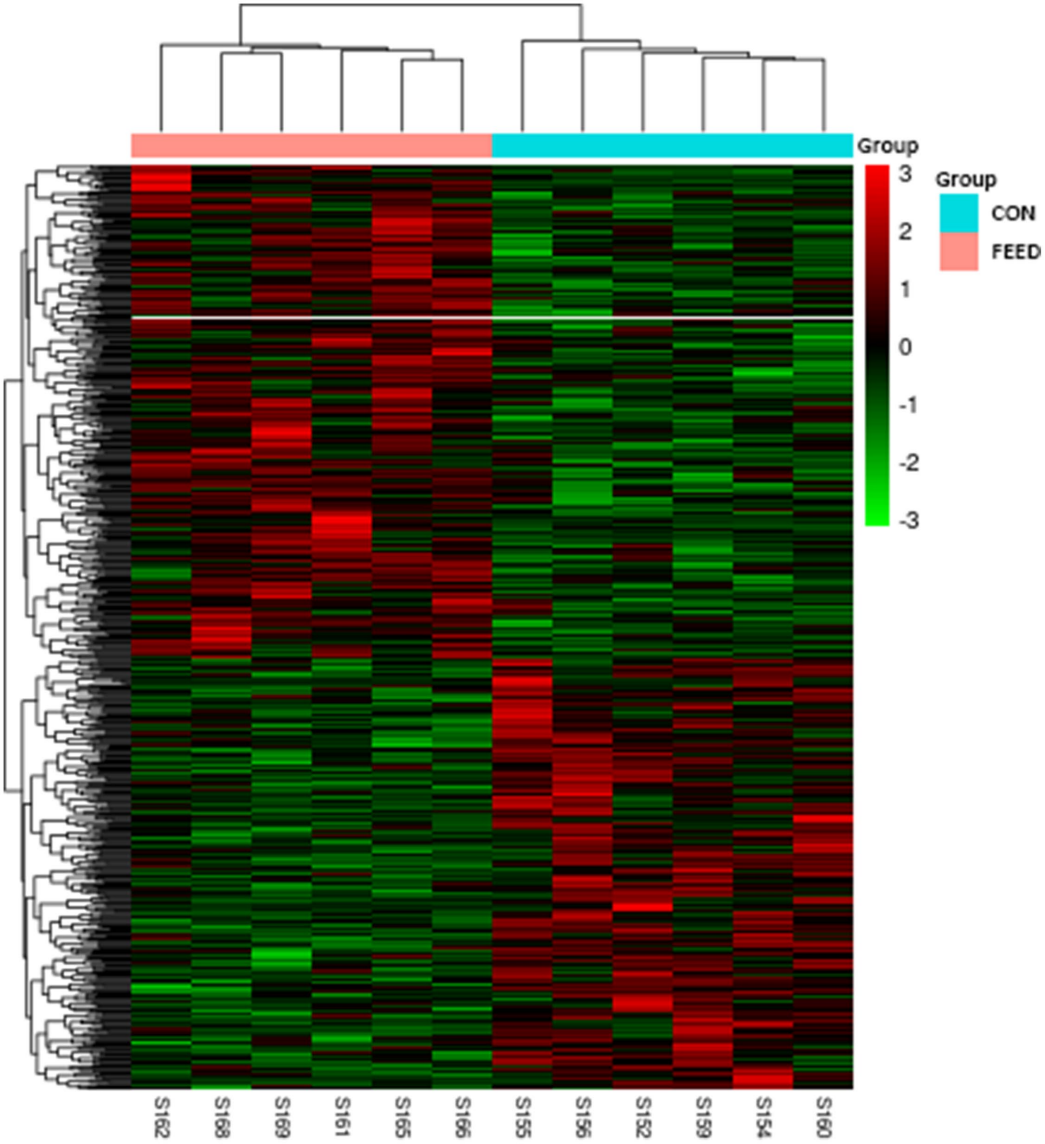
Heatmap with hierarchical clustering analysis for DEG in liver tissues of *Hanwoo* cattle fed with different dietary intervention. Rows represent individual genes, while columns correspond to samples from each group. The color scale indicates the expression levels of the genes, with red representing upregulated genes and green representing downregulated genes. The hierarchical clustering dendrograms on the top and side of the heatmap demonstrate the similarity between samples and genes, respectively, grouping them based on their expression patterns. CON, *Hanwoo* cattle fed a standard diet; DEG, differential gene expression; FEED, *Hanwoo* cattle fed a standard diet supplemented with antioxidants.

As aforementioned, we established the DEG based on *p-*value which resulted in a total of 641 genes for further downstream analyses. A complete list of gene is provided in the Supplementary Table S3. To visualize the distribution of the DEG, the volcano plot was constructed in which 343 genes were upregulated while 298 genes were downregulated in the FEED group (Figure 2). Next, the heatmap shows the hierarchical clustering of the DEG, providing a visual representation of the expression patterns across samples (Figure 3). As shown, the heatmap reveals distinct clustering of gene expression profiles between the CON and FEED groups, indicating a clear separation in response to antioxidants supplementation. Such results reinforce that although there seem no noticeable changes in phenotypic parameters, biological impacts must have been elicited at a transcriptional level. The combined analysis of the volcano plot and heatmap suggests that the treatment induces significant changes in gene expression that warrants further bioinformatics analyses. However, it is important to note that the threshold applied for DEG identification in this study, |log2(fold change)| > 0 with *p*-value ≤0.05, was intentionally set to be inclusive. This liberal approach may result in the inclusion of genes with minimal fold changes, which could potentially limit the biological significance of some findings. Despite this limitation, our objective was to create a robust transcriptomic dataset that captures subtle yet meaningful gene expression changes for comprehensive analysis.

### 3.4 Enrichment analyses: GO terms

Gene GO term enrichment analysis is to highlight the most relevant GO terms (in this study, GO_BP, GO_MF, and GO_CC) associated with a given gene list. Statistically significant terms (*p* < 0.05) enriched the most in the DEG are shown (Tables 2–4). In this, three different methods were applied to calculate a false null hypothesis (i.e., Bonferroni, Benjamini, FDR). As results, in the GO_BP enrichment, no term passed the false null hypothesis; thus, gene count and raw *p*-values were utilized for the GO analysis in which ‘GO:0006357 ~ regulation of transcription from RNA polymerase II promoter,’ ‘GO:0045944 ~ positive regulation of transcription from RNA polymerase II promoter,’ and ‘GO:0010629 ~ negative regulation of gene expression’ were enriched the most (Table 2). In this, although none of the GO_BP was significant after adjusting the FDR (as well as Bonferroni, and Benjamini). Overall, it seems that the intervention of antioxidants impacted genes related RNA polymerase II regulation in the *Hanwoo* cattle liver.

**Table 2:** Gene Ontology (GO) – Biological Process (BP) terms enriched in the liver by the antioxidants intervention.

Term	Count	%	*p*-value	Genes	Fold enrichment	Bonferroni	Benjamini	FDR
GO:0006357 ~ regulation of transcription from RNA polymerase II promoter	45	7.21	0.017	CEBPA, ZNF395, ZNF391, PRDM1, MEOX1, MED15, MECOM, ZNF529, NACC2, ATOH8, ZNF746, HSF4, ANKRD1, ZNF623, MSX1, RXRG, PKNOX2, ZNF286A, TCF7L2, TFAP2B, EGR1, MEF2C, JUND, PRRX1, IRX3, EBF1, MED9, ARNT, FOS, NR4A1, HEYL, MYCN, MAFG, DGKQ, ZNF239, FOSB, NCOA7, PGR, MDM4, ZSCAN26, ZNF215, MKX, MXD1, HMX2, MXD4	1.42	1	1	1
GO:0045944 ~ positive regulation of transcription from RNA polymerase II promoter	29	4.65	0.006	CEBPA, KDM1B, FHOD1, RSF1, MEOX1, PRDM10, FSTL3, RPS6KA4, ZNF746, HSF4, RIPK1, RXRG, TCF7L2, TFAP2B, EGR1, EDN1, MEF2C, AUTS2, SS18L1, IRX3, ARNT, FOS, SMARCA2, BMP6, NR4A1, HEYL, MAFG, NCOA7, MDM4	1.72	0.999967	1	1
GO:0010629 ~ negative regulation of gene expression	14	2.24	0.000	MEF2C, CDKN1A, SLC24A3, PLAG1, PDGFB, PRDM1, NTS, ACACB, CRKL, HEYL, MYCN, DGKQ, ATOH8, PGR	3.51	0.268913	0.313194	0.313194
GO:0045892 ~ negative regulation of transcription, DNA-templated	14	2.24	0.023	CEBPA, TFAP2B, ADIPOQ, RSF1, SMARCA2, CBFA2T3, SCML1, CDK5, MECOM, ATOH8, ZNF746, ANKRD1, MPHOSPH8, BEND5	2.00	1	1	1
GO:0007155 ~ cell adhesion	13	2.08	0.048	RIPOR2, ITGA4, VWF, PCDHGC3, ICAM3, ATP1B1, PCDH18, RELN, ITGAD, PRPH2, COL4A3, BSG, LOC614923	1.86	1	1	1
GO:0006954 ~ inflammatory response	12	1.92	0.022	POLB, CCL25, PTGER4, MFHAS1, FUT7, LOC616364, ACER3, CCL2, CCL1, CD14, LOC112445051, BMP6	2.19	1	1	1
GO:0070374 ~ positive regulation of ERK1 and ERK2 cascade	9	1.44	0.029	CCL25, MFHAS1, GLIPR2, LOC616364, GPR55, PDGFB, CCL2, CCL1, CRKL	2.48	1	1	1
GO:0001764 ~ neuron migration	7	1.12	0.023	RELN, CDK5, DCX, DCDC2, NAV1, NTN1, CRKL	3.17	1	1	1
GO:1902895 ~ positive regulation of pri-miRNA transcription from RNA polymerase II promoter	6	0.96	0.001	EGR1, NGFR, SMAD3, ATOH8, PDGFB, FOS	7.35	0.9	1	1
GO:0071346 ~ cellular response to interferon-gamma	6	0.96	0.034	RAB43, CCL25, LOC616364, CCL2, CCL1, LOC783604	3.33	1	1	1

BP, Biological process; FDR, False discovery rate, GO, Gene ontology.

**Table 3:** Gene Ontology (GO) – Molecular Function (MF) terms enriched in the liver by the antioxidants dietary intervention.

Term	Count	%	*p*-value	Genes	Fold enrichment	Bonferroni	Benjamini	FDR
GO:0042803 ~ protein homodimerization activity	27	4.33	0.0002	CEBPA, MYOM1, TENM1, PDGFB, ATP2A1, MYOM3, JCHAIN, GLIPR2, NACC2, PIP4K2B, RIPK1, TFAP2B, MIGA2, SMAD3, GCH1, IDH1, ADIPOQ, TSC2, ARNT, IRAK3, COQ9, COMMD1, HEYL, PRPH2, MVD, ABCG1, DGKH	2.28	0.08	0.05	0.049353
GO:0000978 ~ RNA polymerase II core promoter proximal region sequence-specific DNA binding	40	6.41	0.0002	CEBPA, ZNF395, PRDM4, PLAG1, ZNF391, PRDM1, MEOX1, MECOM, ZNF529, NACC2, ATOH8, ZNF746, HSF4, ZNF623, RXRG, PKNOX2, ZNF286A, TCF7L2, TFAP2B, EGR1, MEF2C, JUND, SMAD3, PRRX1, IRX3, EBF1, ARNT, FOS, NR4A1, HEYL, MYCN, MAFG, ZNF239, FOSB, PGR, ZSCAN26, ZNF215, MKX, MXD1, MXD4	1.87	0.11	0.05	0.049353
GO:0001228 ~ transcriptional activator activity, RNA polymerase II transcription regulatory region sequence-specific binding	21	3.37	0.0003	CEBPA, TFAP2B, EGR1, MEF2C, ZNF395, JUND, SMAD3, PRRX1, PRDM4, PLAG1, ZNF391, FOS, MEOX1, NR4A1, HEYL, MYCN, MECOM, MAFG, FOSB, PGR, MSX1	2.54	0.14	0.05	0.049353
GO:0000981 ~ RNA polymerase II transcription factor activity, sequence-specific DNA binding	35	5.61	0.0015	CEBPA, PRDM4, PLAG1, MEOX1, ZNF529, NACC2, ATOH8, HSF4, ZNF623, MSX1, PKNOX2, ZNF286A, TCF7L2, TFAP2B, EGR1, MEF2C, JUND, SMAD3, PRRX1, IRX3, EBF1, ARNT, FOS, NR4A1, HEYL, MYCN, MAFG, ZNF239, FOSB, ZSCAN26, ZNF215, MKX, MXD1, HMX2, MXD4	1.76	0.58	0.21	0.213155
GO:0009982 ~ pseudouridine synthase activity	4	0.64	0.0027	RPUSD4, RPUSD1, PUS1, PUSL1	13.84	0.78	0.30	0.301247
GO:0003700 ~ transcription factor activity, sequence-specific DNA binding	13	2.08	0.0050	CEBPA, TFAP2B, MEF2C, ZNF395, JUND, ARNT, PRDM1, FOS, MEOX1, ZNF746, HSF4, FOSB, PGR	2.57	0.94	0.47	0.471462
GO:0016829 ~ lyase activity	4	0.64	0.0066	POLB, PM20D1, PTGES2, CLYBL	10.20	0.98	0.51	0.507939
GO:0050660 ~ flavin adenine dinucleotide binding	6	0.96	0.0078	ACADL, KDM1B, ACOX3, MTO1, ACADS, GFER	4.84	0.99	0.51	0.507939
GO:0005509 ~ calcium ion binding	24	3.85	0.0081	GALNT3, CLSTN2, PCDHGC3, EFCAB14, ATP2A1, PCDH18, HPCAL4, FSTL3, SYT6, NELL2, PRRG4, SMOC2, THBD, PLSCR3, CDH20, MYL1, SPOCK2, ACER3, CDHR2, NCS1, DNER, S100A14, ENPP3, MATN3	1.80	0.99	0.51	0.507939
GO:0003677 ~ DNA binding	29	4.65	0.0107	CEBPA, IGHMBP2, MEOX1, POLB, ANKRD1, SURF6, DTD1, BEND5, PKNOX2, ZBED3, ZNF286A, TCF7L2, TFAP2B, TIGD5, MEF2C, JUND, SMAD3, MBD6, PRRX1, PTGES2, IRX3, ARNT, FOS, SMARCA2, NR4A1, FOSB, PGR, MCM6, PHF19	1.64	1	0.61	0.606762
GO:0031625 ~ ubiquitin protein ligase binding	12	1.92	0.0119	MFHAS1, NGFR, GPI, GABARAPL1, CDKN1A, SMAD3, SLC22A18, ZNF746, RIPK1, TRAF2, AXIN2, FHIT	2.40	1	0.62	0.614162
GO:0008009 ~ chemokine activity	5	0.80	0.0136	CCL25, CXCL12, LOC616364, CCL2, CCL1	5.38	1	0.64	0.639378
GO:0048020 ~ CCR chemokine receptor binding	4	0.64	0.0158	CCL25, LOC616364, CCL2, CCL1	7.45	1	0.69	0.689821
GO:0050840 ~ extracellular matrix binding	4	0.64	0.0176	SMOC2, SPOCK2, BGN, FSTL3	7.18	1	0.71	0.70984
GO:0042802 ~ identical protein binding	32	5.13	0.0196	RIPOR2, CD84, ALAS1, FHOD1, IGHMBP2, ACACB, FHIT, FGFRL1, CRKL, PSTPIP1, HSF4, MLYCD, TOPBP1, CEP55, TIMM8A, SLC14A1, SMURF2, VWF, MX1, ADIPOQ, TRAF2, KCNRG, FOS, TRAIP, NR4A1, MARCKS, USH1G, PRPH2, MAFG, PGR, MCM6, RAB9B	1.52	1	0.74	0.740352
GO:0044877 ~ macromolecular complex binding	8	1.28	0.0242	SCAMP5, CDKN1A, BAG3, NACC2, NUF2, GNB4, RIPK1, TRAF2	2.81	1	0.86	0.857369
GO:0046982 ~ protein heterodimerization activity	14	2.24	0.0273	TFAP2B, TENM1, MEF2C, MIGA2, MTTP, PDGFB, ARNT, IRAK3, FOS, BMP6, NR4A1, MAFG, ABCG1, DGKH	1.97	1	0.87	0.862619
GO:0046872 ~ metal ion binding	46	7.37	0.0274	RBM27, PLAG1, ARL3, ACSM1, GALNT18, FHL3, RSF1, FURIN, PRDM1, ACACB, PRDM10, CLYBL, GGTA1, POLB, HMGCL, HPX, RELN, ZNF529, DPH2, ZNF746, PMPCA, ENPP3, DTD1, ZBED3, ZNF286A, ACVR1, TIMM8A, BRD1, ACE, SMAD3, CPSF3, EBF1, ATP2B3, CBFA2T3, TRAIP, SELENOO, RUFY1, PM20D1, MEX3B, ITGAD, TCN2, MDM4, ZSCAN26, CYTB, RAPSN, PHF19	1.36	1	0.865667	0.862619
GO:0005201 ~ extracellular matrix structural constituent	5	0.80	0.0314	COL15A1, COL13A1, COL4A3, CGN1, COLQ	4.18	1	0.938688	0.935383
GO:0051287 ~ NAD binding	4	0.64	0.0402	ADH4, BDH2, ALDH2, IDH1	5.24	1	1	0.998236
GO:0003951 ~ NAD+ kinase activity	3	0.48	0.0469	DGKQ, NADK, DGKH	8.55	1	1	0.998236

FDR, False discovery rate; GO, Gene ontology; MF, Molecular function.

**Table 4:** Gene Ontology (GO) – Cellular Compartment (CC) terms enriched in the liver by the antioxidants dietary intervention.

Term	Count	%	*p*-value	Genes	Fold enrichment	Bonferroni	Benjamini	FDR
GO:0005739 ~ mitochondrion	42	6.73	0.000	ALAS1, YJEFN3, ACSM1, RAP1GDS1, ATP2A1, ALDH1L2, ACACB, MTO1, FHIT, BPHL, HMGCL, PRDX5, KLC3, ALDH2, ACADL, NACC2, RIPK1, PMPCA, PUSL1, S1PR4, ACADS, GABARAPL1, DGAT2, PTGES2, IDH1, PUS1, CHCHD10, ATAD3A, GFER, RPUSD4, SELENOO, NR4A1, MSRA, PLSCR3, LOC783202, ATG4B, CYTB, NAXD, MXD1, ND5, ABCG1, ND4	2.17	0.00	0.00	0.00
GO:0005654 ~ nucleoplasm	72	11.54	0.000	GPI, ALAS1, FHOD1, RSF1, ALDH1L2, MED15, CRKL, RPS6KA4, NUDCD1, NUF2, NUP62, SPIN1, ANKRD1, PIP4K2B, KPNA5, KPNA2, MAF1, ACADS, PKNOX2, DENND2C, C3H1ORF52, NFKBIL1, PUS1, VWA5A, VPS37A, COMMD1, FOS, CBFA2T3, TRAIP, RPUSD4, MYCN, BLVRB, CCDC86, ZSCAN26, MCM6, MGLL, FKBP5, ARL3, CDCA8, FSTL3, ADH4, ATOH8, ZNF746, SURF6, MSX1, JAZF1, HEXIM2, TCF7L2, EGR1, NGFR, SMAD3, MBD6, JUND, GCH1, PRRX1, CEP152, SS18L1, NEK7, DCDC2, CDC7, SMARCA2, NR4A1, NEDD1, MSRA, MEX3B, CDK5, FOSB, PSMG1, DZIP1, MXD1, MPHOSPH8, COPS9	1.62	0.01	0.01	0.01
GO:0005737 ~ cytoplasm	106	16.99	0.001	IPO13, RIPOR2, PRDM4, LOC508646, FHOD1, PRDM1, RPS6KA4, IPO8, ANPEP, KPNA5, MAF1, PKNOX2, MEF2C, STARD9, TSC2, SRCIN1, RUFY1, CLIP2, PGR, FKBP5, ANP32A, ARL3, HPN, FHIT, ADH4, FAM221A, PRDX5, KLC3, BAG3, ADGRG6, ISOC1, APOD, TSNAXIP1, GTF2A1L, HEXIM2, EGR1, NGFR, AFAP1L1, SMAD3, SMURF2, KLHL25, IDH1, NEK7, IFI44, CDC7, NR4A1, MFHAS1, MSRA, CDK5, PRPH2, ADI1, GNB4, TUBGCP6, PSMG1, DZIP1, COPS9, SRXN1, BICDL1, MEOX1, POLB, PSTPIP1, NUDCD1, SLC22A18, NCS1, NUP62, CEP55, EDN1, RIPK4, ARNT, IRAK3, AXIN2, COMMD1, SERPINB8, IQCG, BDH2, CNKSR3, CDHR2, DCX, ATG4B, GLCCI1, UMPS, GAS7, AHNAK, CCDC69, IGHMBP2, NTN1, RELN, ATOH8, ZNF746, POC1A, IGF2BP2, SMNDC1, RXRG, S1PR4, DTD1, PTPN18, GCH1, IRX3, MX1, NELL2, NEDD1, HEYL, MARCKS, USH1G, CSDC2,	1.36	0.17	0.06	0.06
GO:0005634 ~ nucleus	108	17.31	0.002	IPO13, PRDM4, KDM1B, GEMIN2, PRDM1, KPNA5, KPNA2, MAF1, PKNOX2, PPP1R16B, MEF2C, NFKBIL1, PUS1, STARD9, EBF1, TSC2, JMJD8, CLIP2, XRN1, CCDC86, SNRPE, PGR, ZSCAN26, ZNF395, ANP32A, ARL3, ZNF391, ACACB, PRDM10, FHIT, FAM221A, PRDX5, BAG3, HEXIM2, EGR1, TFAP2B, SMAD3, JUND, SS18L1, CHCHD10, CDC7, SMARCA2, LSM3, NR4A1, CDK5, ADI1, MAFG, MDM4, MKX, PHF19, COPS9, RBM27, FHL3, MEOX1, POLB, SCML1, NUDCD1, MECOM, ZNF529, SPIN1, ANKRD1, RBM6, BRD1, TIGD5, ARNT, IRAK3, AXIN2, COMMD1, FOS, SENP2, CBFA2T3, RPUSD4, PLSCR3, NCOA7, MCM6, UMPS, HMX2, CEBPA, AHNAK, CCDC69, RAP1GDS1, IGHMBP2, FSTL3, RBM15B, PPP1R7, ATOH8, ZNF746, HSF4, ZNF623, IGF2BP2, MSX1, SMNDC1, DTD1, ZNF286A, JAZF1, TCF7L2, PTPN18, MBD6, PRRX1, PTGES2, IRX3, MX1, ELP6, HEYL, FOSB, ZNF215, PABPC1L, RBM46	1.31	0.45	0.15	0.15
GO:0070469 ~ respiratory chain	4	0.64	0.005	ND4L, CYTB, ND5, ND4	11.41	0.79	0.31	0.31
GO:0005581 ~ collagen trimer	6	0.96	0.006	COL15A1, COL13A1, ADIPOQ, COL4A3, CGN1, COLQ	5.19	0.86	0.32	0.32
GO:0005829 ~ cytosol	79	12.66	0.007	GPI, CDKN1A, ALAS1, FHOD1, GEMIN2, AKR1B1, LCLAT1, CRKL, ANKRD9, RPS6KA4, IPO8, NUDCD1, CLEC5A, NUF2, SPIN1, ANKRD1, KPNA5, ARHGEF40, KPNA2, GABARAPL1, NFKBIL1, VPS37A, TRAF2, COMMD1, FOS, SENP2, SERPINB8, ADRA2B, IQCG, RUFY1, BDH2, PLSCR3, XRN1, KIF16B, DGKQ, BLVRB, ATG4B, PFDN1, MVD, SNRPE, ZSCAN26, RAPSN, ZNF395, AHNAK, PLAG1, RAP1GDS1, ECSCR, FHIT, ADH4, PRDX5, BAG3, ZNF746, NPHP4, IGF2BP2, JAZF1, AFAP1L1, SMAD3, GCH1, PRRX1, PTGES2, IDH1, SS18L1, OSBPL3, MX1, DCDC2, CABLES1, ELP6, NR4A1, NEDD1, MSRA, MEX3B, CDK5, SNX15, PSMG1, PABPC1L, DZIP1, MXD1, MPHOSPH8, RAB9B	1.32	0.91	0.33	0.32
GO:0005759 ~ mitochondrial matrix	10	1.60	0.008	RPUSD4, HMGCL, ALAS1, ALDH2, ACADL, MARS2, ACSM1, PMPCA, MLYCD, ACADS	2.90	0.93	0.33	0.32
GO:0009986 ~ cell surface	16	2.56	0.011	NGFR, ITGA4, CLSTN2, HHIP, HPN, ADIPOQ, PDGFB, BGN, FURIN, ADRA2B, LOC112445051, CLEC5A, ADGRG6, GPC3, SLITRK6, GPC4	2.07	0.98	0.42	0.41
GO:0005782 ~ peroxisomal matrix	3	0.48	0.021	PRDX5, MLYCD, HAO2	13.22	1.00	0.68	0.67
GO:0031012 ~ extracellular matrix	10	1.60	0.024	ADAMTSL1, COL15A1, RELN, COL13A1, VWF, LRRTM3, COL4A3, CGN1, BGN, COLQ	2.41	1.00	0.68	0.67
GO:0005777 ~ peroxisome	6	0.96	0.025	HMGCL, PRDX5, IDH1, ACOX3, HAO2, ACOT4	3.64	1.00	0.68	0.67
GO:0005615 ~ extracellular space	39	6.25	0.031	GPI, COL15A1, DEFB7, LOC100847119, COL13A1, MTCL1, PDGFB, LOC112445051, TSKU, FSTL3, HPX, RELN, LRRTM3, GLIPR2, LOC616364, CGN1, CCL2, APOD, CCL1, S100A14, ZBED3, CCL25, EDN1, VWF, TFPI2, ADIPOQ, BGN, SERPINB8, BMP6, COLQ, PM20D1, SMOC2, CXCL12, TCN2, COL4A3, CATHL2, CATHL1, PI15, COL6A5	1.40	1.00	0.73	0.72
GO:0031594 ~ neuromuscular junction	5	0.80	0.031	CDK5, CHRNE, COLQ, RAPSN, CRKL	4.18	1.00	0.73	0.72
GO:0005576 ~ extracellular region	26	4.17	0.033	ITIH3, FURIN, A1BG, NTS, NTN1, PRRG4, ISLR, RELN, GLIPR2, SPOCK2, CD14, ENPP3, MSMB, ACE, VWF, RNASET2, ENTPD5, ADIPOQ, BGN, BMP6, NELL2, SMOC2, BRB, TCN2, CLCF1, CATHL1	1.54	1.00	0.73	0.72
GO:0014069 ~ postsynaptic density	7	1.12	0.048	NGFR, CDK5, DLG5, NCS1, MX1, TSC2, SRCIN1	2.67	1.00	0.99	0.97

CC, Cellular compartment; FDR, False discovery rate; GO, Gene ontology.

Second, GO_MF was explored where a few MF were significant after adjusting FRD. Specifically, ‘GO:0042803 ~ protein homodimerization activity,’ ‘GO:0000978 ~ RNA polymerase II core promoter proximal region sequence-specific DNA binding,’ and ‘GO:0001228 ~ transcriptional activator activity, RNA polymerase II transcription regulatory region sequence-specific binding’ were significantly enriched in the DEG after the intervention. Similar to the GO_BP, the GO_MF results indicate that the antioxidant intervention might have influenced the transcriptional regulation (GO:0000978l and GO:0001228; Table 3). On the other hand, specifically looking at the genes related to the ‘GO:0042803,’ a few key biological functions might be impacted by the intervention such as signal transduction/transcription regulation (SMAD3, ANRT, HEYL, and TRAP2B), metabolic processes (IDH1, GCH1, TSC2, and MVD), and cell survival (RIPK1, and IRAK3; Table 3).

Lastly, GO_CC was investigated to find if specific cellular compartments are impacted compared to the others. As shown in the Table 4, the GO:0005739 ~ mitochondrion was enriched the most with the fold enrichment of 2.17 followed by nucleoplasm and cytoplasm all of which were statistically significant after FDR adjustments (Table 4). With the list of genes enriched in the GO:0005739 ~ mitochondrion, one can expect that multiple biological functions might be impacted by the intervention in the liver mitochondria including mitochondrial dynamics and lipid metabolism. Specifically, ATAD3A gene encodes a mitochondrial membrane protein involved in a wide array of processes including mitochondrial dynamics, nucleoid organization, and cholesterol metabolism and mutations in ATAD3A can lead to neurological disorders by disrupting mitochondrial structure, leading to increased mitophagy and impaired energy production (8, 9). Similarly, RIPK1 is known for its role in regulating cell death, particularly necroptosis, RIPK1 also influences mitochondrial function by modulating reactive oxygen species (ROS) production (10, 11). In contrast, dysregulation of RIPK1 can result in excessive oxidative stress, leading to mitochondrial dysfunction. Interestingly, in our condition, the antioxidant intervention has increased gene expression of mitochondrial RIPK1 (≈120% increase compared to the CON group; *p*-value = 0.019; Supplementary Table S3). In addition, lipid metabolism is crucial for energy storage, membrane synthesis, and signaling (12). Specifically, ACACB gene encodes an enzyme involved in fatty acid metabolism by regulating the conversion of acetyl-CoA to malonyl-CoA, a key step in fatty acid biosynthesis (13). In our DEG list, the ACACB gene was increased in the FEED group compared to the CON group (≈158% increase compared to the CON group; *p*-value = 0.035; Supplementary Table S3). Nonetheless, overall changes in specific genes shown at the hepatic RNA level were not aligned with muscle phenotypes such as intramuscular fat content (Figure 1).

Likewise, the gene ELOVL5 expression was increased in the FEED group compared to the CON group (≈160% increase compared to the CON group; *p*-value = 0.001; Supplementary Table S3); the gene is involved in the elongation of long-chain fatty acids. This gene plays a crucial role in fatty acid metabolism by extending the carbon chain length of polyunsaturated fatty acids, which are important for lipid biosynthesis. Specifically, ELOVL is essential for producing long-chain fatty acids, which contribute to cell membrane composition, signaling, and lipid storage (14). In livestock, the ELOVL gene, which is activated by transcription factors including KLFs, have been associated with differences in intramuscular fat content and overall meat quality due to its impact on lipid metabolism (15). Again, however those specific genetic markers were not able to fully explain the phenotypic characteristics of *Hanwoo* beef cattle.

### 3.5 Functional annotation clustering

**Figure 4:**
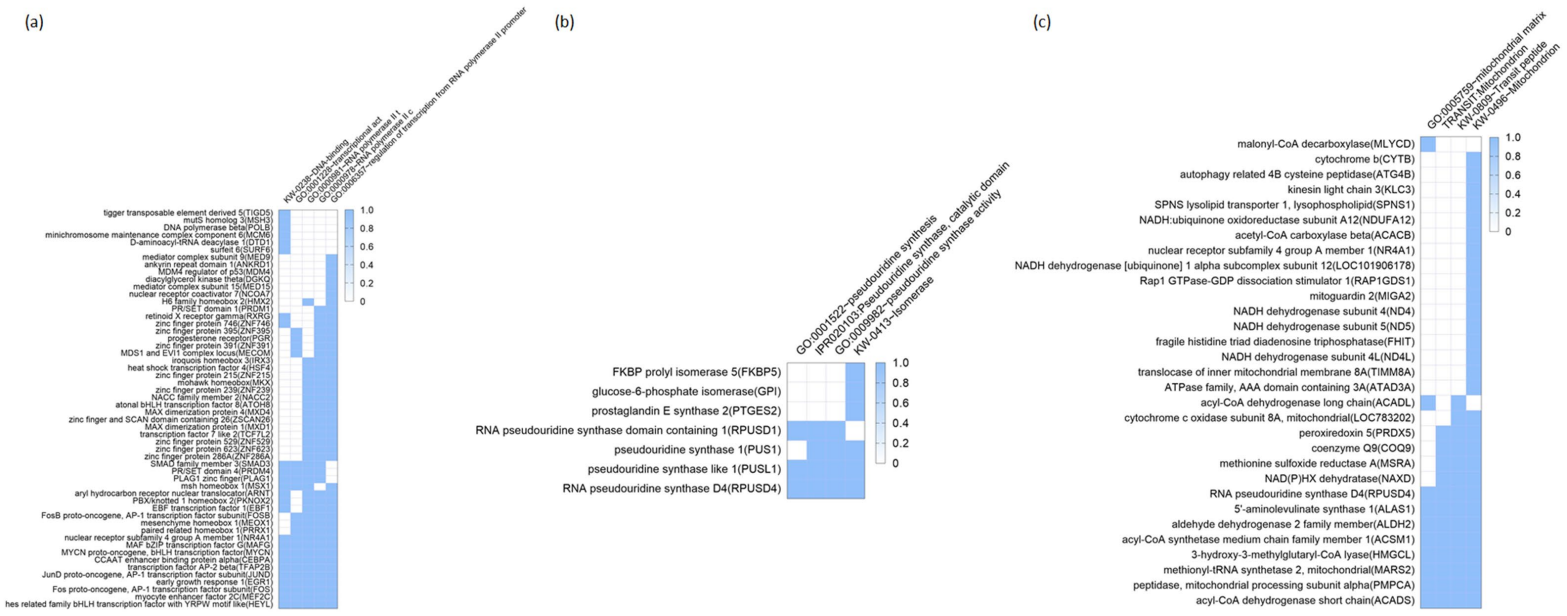
Functional annotation cluster enriched by antioxidant intervention. Heatmaps represent the top three clusters with the highest enrichment scores **(a–c)**: **(a)** 1st cluster (enrichment score: 2.86), **(b)** 2nd cluster (enrichment score: 1.97), and **(c)** 3rd cluster (enrichment score: 1.76). Rows represent individual genes, and columns correspond to specific biological processes or molecular functions, as identified by Gene Ontology (GO) terms. The color intensity reflects the degree of enrichment, with darker blue shades indicating higher enrichment scores. GO, Gene Ontology.

Additionally, functional annotation clustering was carried out which reports groups similar annotations together. The grouping algorithm is based on the hypothesis, and similar annotations should have similar gene members; the more common genes annotations share, the higher chance they will be grouped together. The enrichment score, the geometric mean (in-log scale) of members’ *p*-values in a corresponding annotation cluster, is used to rank their biological significance. Thus, the top-ranked annotation groups most likely have lower *p*-values for their annotation members. Here we provide heat maps of the clusters (i.e., clusters with the top 3 highest enrichment scores; Figures 4a–c). In the first cluster with the highest enrichment score (2.86), included terms were ‘GO:0000978,’ ‘GO:0001228,’ ‘GO:0000981,’ ‘KW-0283,’ and ‘GO:0006357,’ all of which were related with transcription regulation (Figure 4a). This enrichment in transcription regulation is particularly significant in the liver, a central organ for metabolism and homeostasis. Hepatic transcriptional regulation governs the expression of genes involved in critical processes such as lipid metabolism, detoxification, and energy production. For instance, disruptions in transcription factors, including those related to RNA polymerase II activity, can lead to metabolic disorders and impaired liver function (16). These results suggest that the observed transcriptional changes may have broad implications for the metabolic and functional adaptation of the liver under antioxidant supplementation. The second cluster (with its enrichment score of 1.97) includes the terms ‘IPR020103,’ ‘GO:0009982,’ ‘GO:0001522,’ and ‘KW-0413’ (Figure 4b); as shown, the terms were closely related to pseudouridine synthesis which is a critical step in RNA molecule processes (e.g., tRNA, or rRNA) (17). Disruptions in pseudouridine synthesis have been linked to various metabolic disorders, including those affecting lipid metabolism and protein synthesis, which can lead to impaired liver function (18). Therefore, the observed regulation of pseudouridine synthesis in this study may reflect an adaptive response to oxidative stress and nutritional interventions in liver cells. Lastly, the third cluster (enrichment score: 1.76) focuses on terms related to mitochondrial functions: ‘KW-0496,’ ‘GO:0005759,’ ‘TRANSIT: Mitochondrion,’ and ‘KW-0809’; the above terms included genes related to NADH/NADPH recycle, and energy metabolism (Figure 4c). Mitochondrial functions are crucial in the liver for energy metabolism and oxidative stress regulation (12, 19). Efficient NADH/NADPH recycling and energy metabolism maintain liver homeostasis, and disruptions in these processes can impair liver function (20, 21). This underscores the importance of mitochondrial regulation in liver health and metabolic balance, suggesting that antioxidant supplementation affects mitochondrial functions in *Hanwoo* cattle liver. The other clusters were provided in the Supplementary Table S4.

### 3.6 Identification of potential pathway by the intervention: KEGG pathway analysis

Using the KEGG database, we identified several candidate pathways that had been enriched in our DEG list. First, ‘bta01100: Metabolic pathways (*Bos taurus*)’ was identified as the first pathway with *p*-value of 0.0017. In this broad pathway, our DEG list included a total of 56 genes which present 8.9% of total genes (Fold enrichment: 1.48; Supplementary Table S5). Other KEGG pathways enriched by the DEG list were ‘bta04010: MAPK signaling pathway,’ and ‘bta04621: NOD-like receptor signaling pathway’ with 14 genes and 10 genes counted, respectively (Supplementary Table S5).

Overall, this study is significant as it fills a gap in the current understanding of *Hanwoo* cattle by providing the first comprehensive RNA-seq dataset focusing on the effects of antioxidant supplementation. Previous research has largely overlooked transcriptomic analyses in *Hanwoo*, especially in the context of dietary interventions. By offering this detailed RNA-seq data, the study contributes valuable insights that can enhance our knowledge of *Hanwoo* cattle biology, particularly in terms of how these animals respond at the molecular level to specific antioxidant interventions. While no significant differences were observed in phenotypic traits such as weight gain, feed conversion ratio, or meat quality, RNA sequencing revealed substantial gene expression changes. Key genes related to mitochondrial function, lipid metabolism, and transcription regulation, such as RIPK1, ACACB, and ELOVL5 genes, were significantly upregulated in the antioxidant-supplemented group. These findings suggest that while the immediate phenotypic impacts may be limited, significant transcriptional responses occur, which could have long-term implications for cellular function and metabolism. The study’s use of robust bioinformatics analyses, including GO term enrichment and KEGG pathway analysis, further supported these observations by highlighting relevant biological pathways such as metabolic processes and MAPK signaling. However, our research also presents some limitations, including the lack of observed phenotypic changes, a relatively small sample size that may limit the generalizability of the findings, and the short-term nature of the observations, which may not fully capture the long-term impacts of the supplementation. Despite these limitations, the study is a valuable contribution, providing important RNA-seq data that can broaden our understanding of the *Hanwoo* breed and offer a foundation for future research in this area.

## Data Availability

The original contributions presented in this study are publicly available in the NCBI Sequence Read Archive (SRA) under BioProject accession number PRJNA1247235 (https://www.ncbi.nlm.nih.gov/bioproject/1247235). In addition, key data supporting the findings of this study are included in the article and its Supplementary material. Further inquiries can be directed to the corresponding author.
