# Supplementary material for: Transcriptomic signatures in response to antioxidants supplementation in Korean cattle beef, Hanwoo: a 7-month feeding study
Source: Front Vet Sci. 2025 Apr 24;12:1546248. doi: 10.3389/fvets.2025.1546248 (PMC12061023; doi:10.3389/fvets.2025.1546248)
Supplement: Supplementary file 1 [file Data_Sheet_1.zip › Supplementary Data Sheet 1/Table _1.DOCX]

**Supplementary files**

**
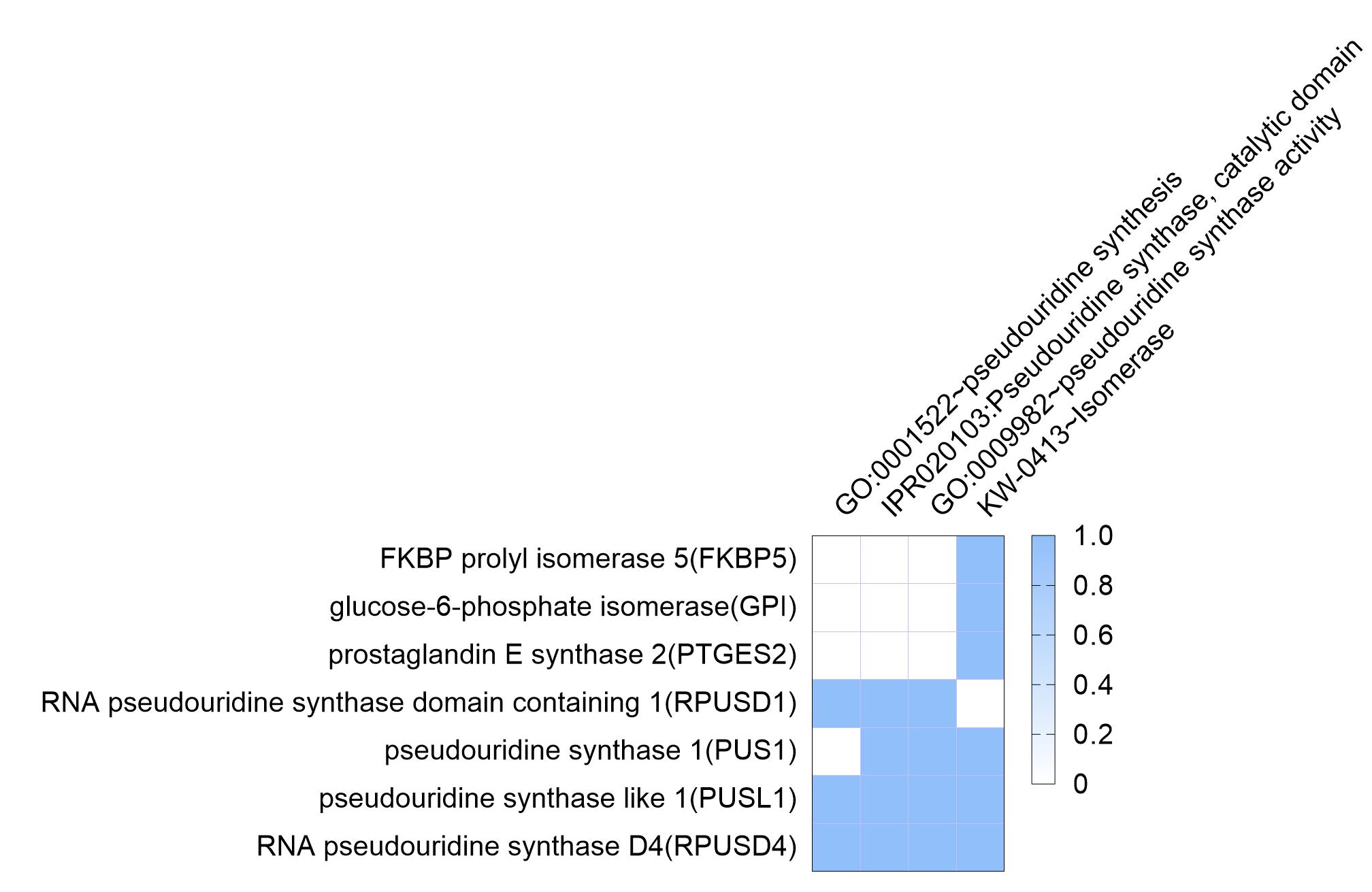
**

**Figure S1. Functional annotation cluster enriched the second most by dietary intervention.** Rows represent individual genes. The cluster includes various genes (rows) associated with specific biological processes or molecular functions (columns), as identified by GO terms. The color intensity represents the degree of enrichment, with darker blue shades indicating higher enrichment scores.


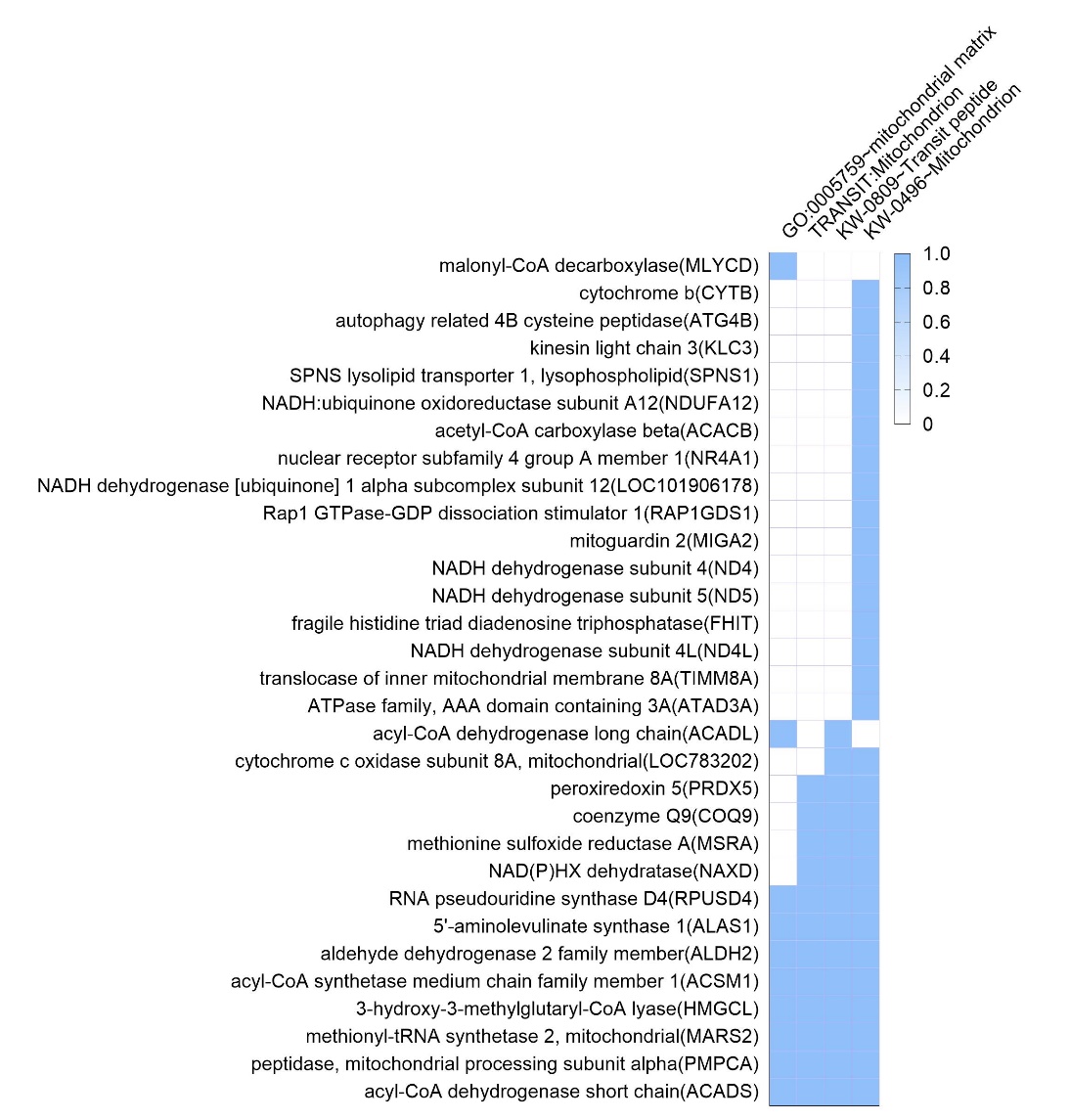


**Figure S2. Functional annotation cluster enriched the third most by dietary intervention.** Rows represent individual genes. The cluster includes various genes (rows) associated with specific biological processes or molecular functions (columns), as identified by GO terms. The color intensity represents the degree of enrichment, with darker blue shades indicating higher enrichment scores.

**
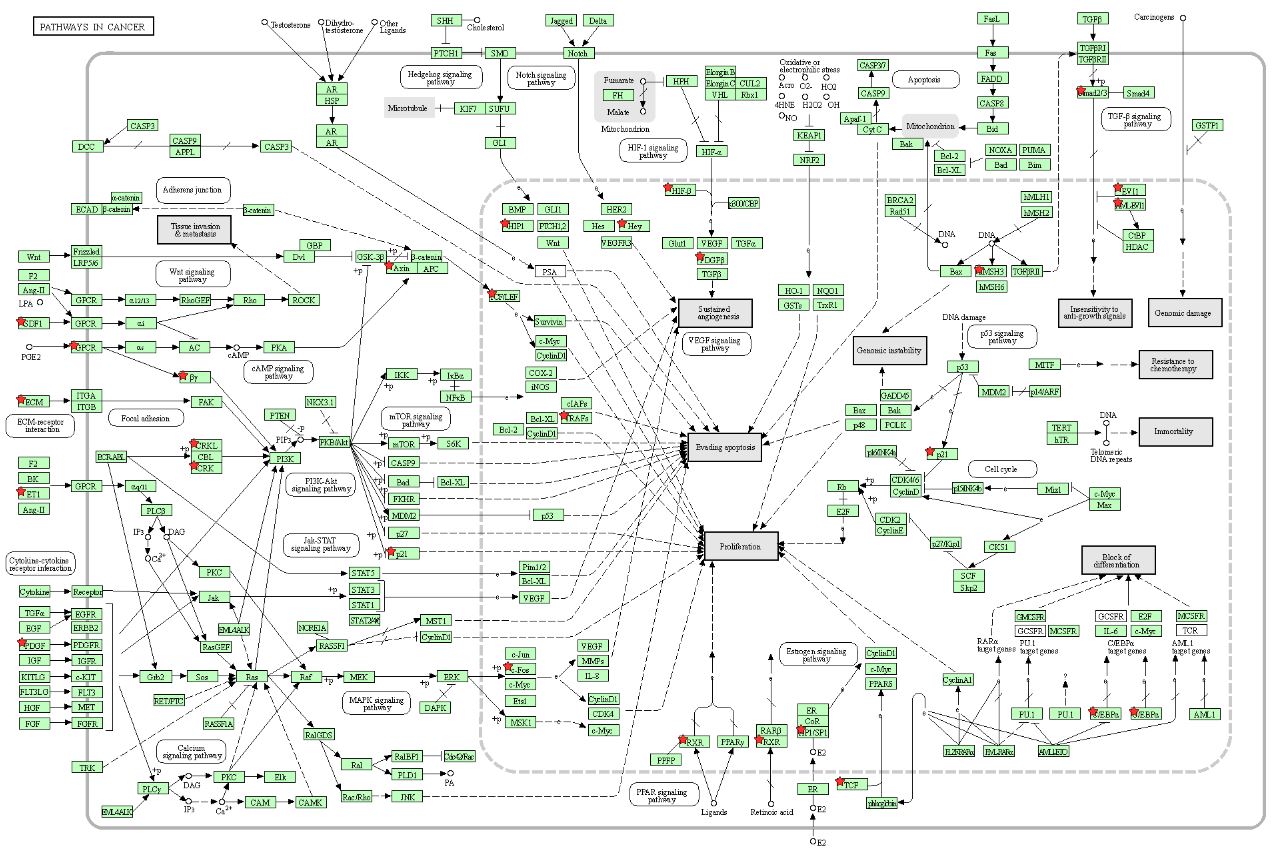
**

**Figure S3.** **The KEGG network analysis present a Pathways in cancer enriched the most by the dietary intervention.** Nodes represent individual genes or proteins within the pathway, while edges indicate interactions between them.


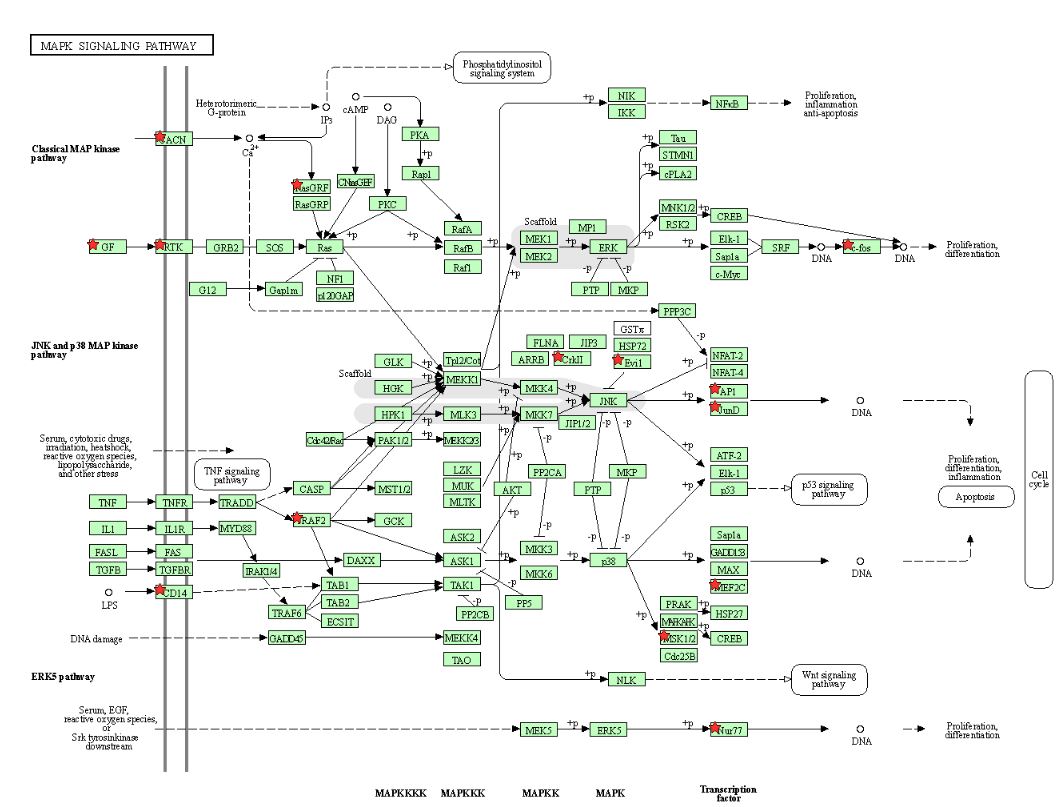


**Figure S4. The KEGG network analysis present a MAPK signaling pathway enriched the second most by the dietary intervention.** Nodes represent individual genes or proteins within the pathway, while edges indicate interactions between them.

| No | Sample Name | Concentration (ng/ul) | RIN | Purity  260/230 | Purity  260/280 | Sample QC Results |
| --- | --- | --- | --- | --- | --- | --- |
| 1 | CON-S152 | 2193.12 | 7.3 | 1.62 | 2.08 | PASS |
| 2 | CON-S154 | 2573.59 | 7.5 | 1.71 | 2.06 | PASS |
| 3 | CON-S155 | 1517.65 | 7.4 | 1.53 | 2.11 | PASS |
| 4 | CON-S156 | 1227.09 | 7.6 | 1.66 | 2.08 | PASS |
| 5 | CON-S159 | 1497.42 | 7.4 | 1.84 | 2.10 | PASS |
| 6 | CON-S160 | 1854.83 | 7.5 | 1.42 | 2.09 | PASS |
| 7 | EXP-S161 | 1757.15 | 7.3 | 1.84 | 2.09 | PASS |
| 8 | EXP-S162 | 3003.28 | 7.4 | 1.73 | 2.04 | PASS |
| 9 | EXP-S165 | 1133.59 | 7.4 | 1.51 | 2.07 | PASS |
| 10 | EXP-S166 | 1301.59 | 7.5 | 1.60 | 2.09 | PASS |
| 11 | EXP-S168 | 1828.12 | 7.6 | 1.73 | 2.09 | PASS |
| 12 | EXP-S169 | 2193.12 | 7.6 | 1.83 | 2.09 | PASS |

**Table S1.** **RNA quality control**

**Table S2. Trimmed sequencing data quality control**

| Sample Name | Total reads | Overall mapped read, % | Uniquely  mapped read, % | Q30, % | GC, % |
| --- | --- | --- | --- | --- | --- |
| CON-S152 | 50636942 | 49393913(97.55%) | 47820439(94.44%) | 93.56 | 49.36 |
| CON-S154 | 47827274 | 46565450(97.36%) | 44926565(93.94%) | 93.52 | 49.18 |
| CON-S155 | 47044536 | 45906169(97.58%) | 44253971(94.07%) | 93.51 | 49.72 |
| CON-S156 | 41533728 | 40593535(97.74%) | 39313898(94.66%) | 93.93 | 49.25 |
| CON-S159 | 49576368 | 48407185(97.64%) | 46727169(94.25%) | 93.74 | 49.42 |
| CON-S160 | 59277244 | 57840422(97.58%) | 55763272(94.07%) | 93.32 | 49.3 |
| FEED-S161 | 62888644 | 61404596(97.64%) | 59251939(94.22%) | 94.47 | 49.42 |
| FEED-S162 | 56271120 | 54845849(97.47%) | 53074948(94.32%) | 94.65 | 49.36 |
| FEED-S165 | 55136164 | 53590457(97.2%) | 51928337(94.18%) | 93.87 | 49.75 |
| FEED-S166 | 54130536 | 52772413(97.49%) | 50957458(94.14%) | 94.68 | 49.93 |
| FEED-S168 | 54148298 | 52807483(97.52%) | 50949417(94.09%) | 94.31 | 49.3 |
| FEED-S169 | 50116912 | 48946583(97.66%) | 47169119(94.12%) | 94.55 | 49.56 |

**Table S3. DEG list in liver tissues of Hanwoo cattle fed with different dietary intervention**

| **Gene_name** | **FEED_GROUP** | **CON_GROUP** | **log2FoldChange** | **pvalue** |
| --- | --- | --- | --- | --- |
| ADIPOQ | 87.73997436 | 0.556124023 | 7.313802 | 0.006286 |
| LOC112448801 | 107.4529569 | 2.153498462 | 5.616739 | 9.39E-09 |
| MYL1 | 10.44449622 | 0.50906159 | 4.319316 | 0.001425 |
| LOC107131601 | 4.763940206 | 0.196456219 | 4.14706 | 0.000168 |
| LOC112442060 | 6.248850294 | 0.369276779 | 4.051379 | 0.014297 |
| CCDC81 | 2.66498397 | 0 | 3.774483 | 0.022586 |
| OR11H6 | 10.23490547 | 0.756686615 | 3.679121 | 0.006677 |
| ST8SIA1 | 2.398084367 | 0 | 3.630531 | 0.037416 |
| LOC112448893 | 3.347379248 | 0.182429536 | 3.629061 | 0.005774 |
| LOC101902321 | 3.218350294 | 0.186847243 | 3.571318 | 0.00596 |
| LOC508646 | 2.267691748 | 0 | 3.548918 | 0.042868 |
| LOC101902555 | 9.380382165 | 0.89552883 | 3.415369 | 0.001088 |
| SHC3 | 2.811799431 | 0.171566631 | 3.386957 | 0.008221 |
| LOC112442317 | 1.971320889 | 0 | 3.348461 | 0.024737 |
| C23H6orf222 | 1.96660575 | 0 | 3.347427 | 0.01288 |
| USH1G | 3.421894854 | 0.326632053 | 3.18438 | 0.021693 |
| CLSTN2 | 4.692781689 | 0.523009808 | 3.146695 | 0.012 |
| MATN3 | 2.325653551 | 0.196456219 | 3.098858 | 0.019758 |
| LOC101906664 | 3.093703784 | 0.33104976 | 3.031429 | 0.038073 |
| SPATA22 | 4.15972317 | 0.468281581 | 3.019026 | 0.010039 |
| LOC112445051 | 3.006081533 | 0.351443177 | 3.000515 | 0.012242 |
| PGR | 4.158721965 | 0.531319724 | 2.960756 | 0.049154 |
| LOC112444774 | 2.946368126 | 0.324079064 | 2.958542 | 0.00967 |
| LOC112443432 | 2.011455619 | 0.186847243 | 2.890536 | 0.048533 |
| LOC112449031 | 30.41788682 | 4.181478492 | 2.861718 | 0.013467 |
| LOC112448396 | 2.701224025 | 0.353996167 | 2.842086 | 0.047975 |
| ZNF215 | 11.43211203 | 1.645556083 | 2.836336 | 0.00042 |
| GTF2A1L | 2.675297029 | 0.288405034 | 2.815529 | 0.037383 |
| ANKDD1B | 3.699686279 | 0.54084341 | 2.800765 | 0.011436 |
| LOC112442644 | 11.16776427 | 1.621440056 | 2.790954 | 0.012901 |
| LOC104969916 | 1.850214713 | 0.182429536 | 2.769596 | 0.040483 |
| KCNJ16 | 4.657515028 | 0.727690653 | 2.721615 | 0.008963 |
| LOC104969678 | 3.474367678 | 0.538290421 | 2.715998 | 0.018136 |
| LOC112442387 | 2.490149473 | 0.373694486 | 2.715196 | 0.044136 |
| LOC112441666 | 2.430621992 | 0.353996167 | 2.663821 | 0.034264 |
| KCNRG | 3.169862934 | 0.484861254 | 2.63435 | 0.024523 |
| CCR9 | 3.194152169 | 0.52750598 | 2.630501 | 0.0363 |
| NELL2 | 4.230260953 | 0.744355616 | 2.542895 | 0.020534 |
| LOC112444976 | 4.466242148 | 0.833666152 | 2.373105 | 0.00896 |
| LOC104975214 | 3.638155297 | 0.736045701 | 2.35037 | 0.039103 |
| LOC782456 | 16.70668304 | 3.563263074 | 2.233349 | 0.040122 |
| LOC104973920 | 3.20739327 | 0.65890241 | 2.225205 | 0.032208 |
| LOC100847415 | 20.60803259 | 4.518034361 | 2.190467 | 0.015291 |
| HMX2 | 5.514059716 | 1.23870225 | 2.175333 | 0.025808 |
| LOC107133234 | 4.675250559 | 1.08177211 | 2.139887 | 0.027533 |
| NUF2 | 167.3799115 | 41.19808774 | 2.023007 | 0.000785 |
| LOC514457 | 33.487557 | 8.637882448 | 1.948431 | 0.001342 |
| LOC510520 | 4.605321405 | 1.197311454 | 1.918073 | 0.046634 |
| LOC112441494 | 29.09182607 | 8.033287849 | 1.858054 | 0.00378 |
| LOC112443878 | 5.098498728 | 1.41286203 | 1.856865 | 0.020734 |
| LOC100297099 | 5.755204023 | 1.652001281 | 1.848752 | 0.028748 |
| LOC112442048 | 7.77896781 | 2.178044446 | 1.844973 | 0.037548 |
| LOC617463 | 7.508008801 | 2.157865759 | 1.828876 | 0.008866 |
| RTL9 | 12.65773762 | 3.700004612 | 1.764997 | 0.013852 |
| KLC3 | 5.004135707 | 1.42645193 | 1.746208 | 0.049479 |
| LOC112444232 | 5.886592664 | 1.736290146 | 1.746057 | 0.038157 |
| IRX3 | 43.11657532 | 13.42370961 | 1.678115 | 0.006113 |
| ST8SIA5 | 9.605303076 | 3.063625477 | 1.638119 | 0.028327 |
| C3H1orf56 | 9.009284628 | 2.871699743 | 1.616441 | 0.019257 |
| LOC616364 | 13.62670544 | 4.461985925 | 1.609963 | 0.034014 |
| SSTR1 | 4.7425584 | 1.587507148 | 1.587667 | 0.043628 |
| DLG2 | 10.73316562 | 3.624273726 | 1.584778 | 0.01066 |
| LOC509911 | 41.5249887 | 14.43295561 | 1.525611 | 0.003395 |
| LIN7A | 16.26777576 | 5.661663196 | 1.524545 | 0.016478 |
| TRAIP | 7.934413036 | 2.836585112 | 1.490648 | 0.006795 |
| LOC104973224 | 12.49726444 | 4.473322857 | 1.48505 | 0.033084 |
| ATP2B3 | 7.849309565 | 2.900063463 | 1.446075 | 0.032045 |
| LOC112444383 | 9.356506433 | 3.499419017 | 1.417305 | 0.029126 |
| LOC101904303 | 14.66541626 | 5.573529412 | 1.397639 | 0.04404 |
| CDHR2 | 27.46494499 | 10.41742909 | 1.388443 | 0.006967 |
| FHIT | 20.84199926 | 8.377484668 | 1.317065 | 0.037078 |
| LOC112444636 | 8.048911692 | 3.422626237 | 1.265484 | 0.040643 |
| DNER | 18.77953189 | 7.890228335 | 1.250514 | 0.036731 |
| LOC112446795 | 46.32305084 | 19.55216876 | 1.242857 | 0.004949 |
| CCDC69 | 329.543903 | 139.3364045 | 1.242397 | 0.033764 |
| C15H11orf94 | 8.407400958 | 3.526828568 | 1.230431 | 0.048179 |
| MIR2440 | 9.692406322 | 4.061382728 | 1.228512 | 0.031384 |
| BRB | 28.87701402 | 12.51666076 | 1.206625 | 0.021226 |
| TM4SF19 | 14.42261758 | 6.348686314 | 1.201292 | 0.042292 |
| MRO | 13.85062722 | 6.092362752 | 1.179371 | 0.032457 |
| KEH36_t19 | 17.18000213 | 7.691280353 | 1.153841 | 0.029639 |
| MIR2887-1 | 7215.545375 | 3265.285713 | 1.143766 | 1.10E-07 |
| LOC101902207 | 9.081463342 | 4.279239776 | 1.106886 | 0.043649 |
| LOC101905407 | 75.18966573 | 36.12880771 | 1.060678 | 0.010987 |
| ANKRD1 | 20.18171366 | 9.680258416 | 1.060547 | 0.030015 |
| LOC112448390 | 13.56704934 | 6.550183024 | 1.057992 | 0.047508 |
| MKX | 69.87189356 | 33.5962247 | 1.057909 | 0.011778 |
| NPHP4 | 13.97997268 | 7.024480036 | 1.015895 | 0.030908 |
| SMOC2 | 233.8119775 | 118.5204809 | 0.980791 | 0.016679 |
| TMEM200C | 57.5254324 | 29.39483814 | 0.977398 | 0.024637 |
| BEND5 | 23.35196835 | 11.93346144 | 0.973756 | 0.007109 |
| ITGAD | 11.83083625 | 6.12224565 | 0.945228 | 0.029109 |
| PPM1J | 24.54692673 | 12.83510793 | 0.943598 | 0.009893 |
| LOC112447346 | 13.29877832 | 6.956477458 | 0.935088 | 0.020628 |
| LOC112443512 | 19.28774479 | 10.24406995 | 0.927566 | 0.01368 |
| MSX1 | 25.27967494 | 13.65094539 | 0.888339 | 0.047834 |
| ARHGAP22 | 20.422252 | 11.16204378 | 0.887647 | 0.027646 |
| CACNB2 | 103.1324535 | 56.4884998 | 0.872867 | 0.015435 |
| LOC101902124 | 30.38866065 | 16.74910129 | 0.865262 | 0.022276 |
| MYOM1 | 91.05662613 | 50.27442839 | 0.857936 | 0.017823 |
| FRMPD1 | 17.45202063 | 9.711862029 | 0.855751 | 0.04187 |
| LOC104975054 | 13.86263299 | 7.789318657 | 0.841276 | 0.026579 |
| LOC783610 | 23.81809151 | 13.13617588 | 0.840836 | 0.007924 |
| TPBGL | 37.00023597 | 20.66613889 | 0.835161 | 0.007054 |
| LOC112444681 | 41235.01512 | 23473.9639 | 0.812799 | 0.003411 |
| ZNF529 | 25.58651188 | 14.62699583 | 0.80415 | 0.011211 |
| LOC101907513 | 19.19330052 | 10.88283473 | 0.799265 | 0.047707 |
| SYT6 | 30.99771416 | 17.97905341 | 0.792339 | 0.036899 |
| LOC107132853 | 83.62595699 | 48.26163004 | 0.788104 | 0.009704 |
| ENPP3 | 53.61566622 | 31.30685288 | 0.784245 | 0.041782 |
| LOC112442408 | 4620.777035 | 2692.32967 | 0.779205 | 0.000489 |
| CGN1 | 15713.69296 | 9157.810642 | 0.778978 | 0.001683 |
| SERPINB8 | 141.2825424 | 83.12695209 | 0.764897 | 0.029025 |
| MED9 | 304.5729917 | 180.5762487 | 0.753871 | 0.011982 |
| LRRTM3 | 19.71878059 | 11.68226023 | 0.746593 | 0.038918 |
| CLCF1 | 68.14528596 | 40.88076368 | 0.741548 | 0.039487 |
| CYP2J2 | 22.03469585 | 13.13747778 | 0.738325 | 0.039339 |
| AUTS2 | 3553.088689 | 2132.329652 | 0.736493 | 0.003526 |
| CES3 | 18138.85989 | 10897.69572 | 0.735053 | 2.12E-06 |
| LOC112447109 | 41.08679466 | 24.70298731 | 0.729206 | 0.037261 |
| EBF1 | 130.0010691 | 79.05347071 | 0.716454 | 0.011702 |
| PP2D1 | 20.04903533 | 12.0858188 | 0.703823 | 0.049355 |
| TSKU | 11014.57222 | 6764.940091 | 0.703295 | 0.022711 |
| ELOVL5 | 10107.98407 | 6314.298272 | 0.678827 | 0.001249 |
| ALAS1 | 2310.102711 | 1443.148147 | 0.678709 | 0.035052 |
| MIR1291 | 27.50568359 | 17.2069892 | 0.669561 | 0.024684 |
| ACACB | 85.87585935 | 54.01670433 | 0.669082 | 0.034909 |
| ATOH8 | 399.2680726 | 252.1895054 | 0.662486 | 0.047026 |
| COLQ | 27.80118307 | 17.61830275 | 0.658238 | 0.014774 |
| AKR1B1 | 56.32168489 | 35.87204517 | 0.65784 | 0.011546 |
| CHRNE | 193.8009987 | 123.0334843 | 0.654664 | 0.025676 |
| ALDH1L2 | 354.2518769 | 225.7388037 | 0.651882 | 0.046125 |
| RASD1 | 88.40062901 | 56.17558809 | 0.645692 | 0.004486 |
| RIPOR3 | 867.0527826 | 556.2599538 | 0.639312 | 0.000944 |
| SRXN1 | 311.8062135 | 200.6206613 | 0.634926 | 0.000337 |
| LOC100847357 | 58.15193087 | 37.69504032 | 0.634669 | 0.037504 |
| SCRN3 | 141.3732144 | 91.04151791 | 0.633878 | 0.023423 |
| PDGFB | 121.8914934 | 78.9065054 | 0.629544 | 0.005875 |
| CDKN1A | 2148.50782 | 1393.72771 | 0.62421 | 0.015458 |
| HSF4 | 285.4349466 | 186.1952518 | 0.618575 | 0.000115 |
| CD84 | 41.08285024 | 27.01904433 | 0.616574 | 0.031313 |
| GALNT18 | 112.4889416 | 73.40439777 | 0.614829 | 0.017253 |
| H4 | 291.9829619 | 191.3831874 | 0.60922 | 0.023629 |
| C21H14orf180 | 195.8125812 | 128.7319919 | 0.606353 | 0.015067 |
| MRAP | 52.65647386 | 34.73027856 | 0.602263 | 0.011957 |
| LOC615303 | 4102.801166 | 2720.783175 | 0.592523 | 0.017088 |
| BICDL1 | 179.9728126 | 119.4915598 | 0.590818 | 0.03158 |
| RGSL1 | 361.9904432 | 240.3978533 | 0.590487 | 0.021493 |
| AXIN2 | 112.2679902 | 74.54178777 | 0.589573 | 0.00255 |
| FKBP5 | 783.3814211 | 521.5878084 | 0.586367 | 0.024799 |
| CABLES1 | 232.8440031 | 156.3590935 | 0.576104 | 0.00276 |
| FAM229A | 50.07444712 | 33.66108683 | 0.575375 | 0.010212 |
| LOC540707 | 26717.58066 | 17981.6104 | 0.571248 | 0.011318 |
| WIPF3 | 90.1241516 | 61.00932303 | 0.568728 | 0.001914 |
| RCL1 | 5204.988554 | 3530.042469 | 0.560099 | 0.027663 |
| POC1A | 74.26822443 | 50.66487755 | 0.559217 | 0.011915 |
| CEACAM19 | 281.8584834 | 192.0163061 | 0.553546 | 0.043175 |
| LOC100140261 | 15347.68241 | 10490.44912 | 0.548943 | 0.002341 |
| CCDC86 | 788.5553028 | 539.1827514 | 0.547522 | 0.001953 |
| LOC101906200 | 184.72115 | 126.2381182 | 0.546627 | 0.038531 |
| C11H9orf116 | 406.066799 | 278.8325266 | 0.540615 | 0.022954 |
| CHST1 | 84.55674377 | 58.08306058 | 0.535706 | 0.020217 |
| DENND2C | 43.44463974 | 30.07889535 | 0.532633 | 0.014618 |
| C3H1orf52 | 117.1842534 | 80.90495507 | 0.529712 | 0.000516 |
| ACOX3 | 1132.153465 | 790.1598061 | 0.518969 | 0.016759 |
| ICAM3 | 290.6089103 | 203.181389 | 0.516877 | 0.008544 |
| MARS2 | 178.243707 | 124.6269741 | 0.516672 | 0.019837 |
| ISM1 | 191.5828705 | 134.0787106 | 0.514392 | 0.035036 |
| TFPI2 | 291.9148578 | 204.5352013 | 0.514261 | 0.036674 |
| ACADL | 3162.257069 | 2220.185952 | 0.510101 | 0.017499 |
| TOPBP1 | 754.4835863 | 529.8314931 | 0.508943 | 0.00741 |
| HEYL | 445.5913161 | 314.3414399 | 0.504669 | 0.006444 |
| NCS1 | 1301.477914 | 917.259953 | 0.504579 | 0.032657 |
| C29H11orf86 | 2876.070618 | 2030.316992 | 0.50235 | 0.000158 |
| MTCL1 | 83.51713617 | 59.09980231 | 0.501283 | 0.014331 |
| ZNF239 | 104.3352462 | 73.81623279 | 0.500053 | 0.033863 |
| PTGER4 | 88.16227105 | 62.58855385 | 0.499303 | 0.030573 |
| ELP6 | 73.30509316 | 51.78200473 | 0.499141 | 0.017832 |
| TMEM169 | 150.8083809 | 107.445095 | 0.489561 | 0.030108 |
| PUSL1 | 65.19987019 | 46.65897625 | 0.48899 | 0.01239 |
| HAO2 | 5524.796008 | 3937.404373 | 0.488587 | 0.010287 |
| BMP6 | 240.4057031 | 171.5315492 | 0.486306 | 0.029497 |
| PTPN18 | 418.6253083 | 299.3516016 | 0.483407 | 0.04913 |
| GPC4 | 246.0113628 | 175.8210847 | 0.482457 | 0.031178 |
| ADAMTSL1 | 90.07046888 | 64.55791933 | 0.480745 | 0.036383 |
| LOC104976195 | 353.8991301 | 253.7984905 | 0.477895 | 0.010061 |
| ZCCHC24 | 2516.254312 | 1818.242717 | 0.468554 | 0.014353 |
| ANKRD9 | 661.985551 | 479.1878544 | 0.46513 | 5.74E-05 |
| MCM6 | 118.4555927 | 85.93322031 | 0.463594 | 0.041817 |
| ADRA2B | 80.53504464 | 58.50001997 | 0.462418 | 0.02209 |
| LOC784417 | 3907.371269 | 2837.346165 | 0.461797 | 0.020671 |
| BAG3 | 3007.484084 | 2196.354491 | 0.453198 | 0.023513 |
| RASL10B | 586.3288552 | 428.9476899 | 0.451585 | 0.004747 |
| CLYBL | 247.7619237 | 181.0057842 | 0.451164 | 0.010698 |
| NUP62 | 763.6731621 | 560.2519303 | 0.446526 | 0.028202 |
| MARCKS | 273.2198008 | 202.3722983 | 0.434188 | 0.032529 |
| CXCL12 | 2546.52639 | 1889.650677 | 0.430584 | 0.00742 |
| RXRG | 639.4200791 | 474.4058593 | 0.430171 | 0.04067 |
| ATP1B1 | 334.8319754 | 249.3309561 | 0.424209 | 0.049714 |
| NCKIPSD | 90.93707263 | 67.41428937 | 0.423494 | 0.030793 |
| CCDC85C | 699.712377 | 521.9651802 | 0.422972 | 0.048664 |
| SCAMP5 | 145.3923386 | 108.5891627 | 0.4224 | 0.020413 |
| RIPK4 | 242.3826394 | 181.3574274 | 0.418933 | 0.022558 |
| HEXIM2 | 208.0354981 | 156.038337 | 0.413515 | 0.048387 |
| ENTPD5 | 7208.191955 | 5414.945994 | 0.41268 | 0.029806 |
| NEDD1 | 421.5148442 | 317.3028491 | 0.407893 | 0.043757 |
| TMEM185B | 187.0154325 | 141.3072769 | 0.404071 | 0.001184 |
| RIN2 | 368.1705256 | 278.4578804 | 0.403323 | 0.028241 |
| PRR5 | 173.0778055 | 131.5309554 | 0.395123 | 0.023789 |
| NTN1 | 772.8096837 | 587.2309134 | 0.395096 | 0.011737 |
| VIPR1 | 1188.603538 | 905.1295319 | 0.393402 | 0.00165 |
| RPUSD1 | 128.4957423 | 97.84388271 | 0.392067 | 0.03146 |
| CNKSR3 | 170.2787369 | 130.1672748 | 0.386473 | 0.001895 |
| SELENOO | 1076.082452 | 823.6672445 | 0.38601 | 0.01638 |
| RAB43 | 2040.80636 | 1561.570309 | 0.385622 | 0.039738 |
| CEBPA | 1179.633342 | 903.6626516 | 0.38416 | 0.028916 |
| LOC112444893 | 570.0866887 | 437.0401202 | 0.383693 | 0.041415 |
| NFKBIL1 | 198.3137342 | 152.091519 | 0.382452 | 0.040037 |
| GGTA1 | 92.70113509 | 71.22367069 | 0.377428 | 0.043032 |
| NACC2 | 151.0555811 | 116.1310374 | 0.376482 | 0.034467 |
| GPR146 | 255.6672051 | 197.2470824 | 0.374626 | 0.048713 |
| MGLL | 5890.813492 | 4548.980571 | 0.372909 | 0.034458 |
| BDH2 | 1365.897974 | 1056.292844 | 0.370836 | 0.04547 |
| KLHL25 | 1772.982687 | 1372.945684 | 0.368917 | 0.003114 |
| TUSC1 | 250.9260255 | 194.376547 | 0.368851 | 0.010738 |
| ZNF783 | 129.4103112 | 100.3599144 | 0.364891 | 0.0187 |
| BLVRB | 1829.935195 | 1422.139479 | 0.364143 | 0.010102 |
| SURF6 | 260.5997334 | 202.1765195 | 0.363629 | 0.02769 |
| IDH1 | 4344.467187 | 3377.553002 | 0.363317 | 0.014554 |
| PRDM1 | 89.41071325 | 69.56981039 | 0.356994 | 0.026792 |
| FGFRL1 | 3191.319717 | 2493.221965 | 0.355821 | 0.00229 |
| LOC112447359 | 83.71249278 | 65.64007557 | 0.35047 | 0.049441 |
| SMURF2 | 121.1845548 | 95.30408952 | 0.347615 | 0.035323 |
| A1BG | 47526.61726 | 37400.8789 | 0.345662 | 0.002829 |
| KBTBD3 | 88.91661778 | 70.08472754 | 0.345172 | 0.025944 |
| GABARAPL1 | 4184.987111 | 3298.139228 | 0.343636 | 0.025346 |
| KIF16B | 460.4470456 | 362.7089569 | 0.343474 | 0.007063 |
| ACOT4 | 445.7554623 | 352.1268121 | 0.341181 | 0.015641 |
| SLC5A6 | 442.320996 | 349.2444818 | 0.340254 | 0.023647 |
| SS18L1 | 177.683877 | 140.077073 | 0.339578 | 0.017993 |
| CHCHD10 | 4432.61263 | 3504.979706 | 0.338964 | 0.003287 |
| SH3BP2 | 718.5869164 | 568.0528066 | 0.338454 | 0.025673 |
| TMEM204 | 332.3879853 | 262.6953994 | 0.33694 | 0.015525 |
| GPI | 2160.791778 | 1712.053805 | 0.335998 | 0.028981 |
| MAFG | 351.4182288 | 278.268447 | 0.335586 | 0.035905 |
| ATAD3A | 510.9349577 | 405.8384403 | 0.330775 | 0.021511 |
| C26H10orf88 | 150.5375165 | 119.7665102 | 0.330615 | 0.029044 |
| DLG5 | 1586.578966 | 1261.655915 | 0.330536 | 0.036893 |
| JUND | 2378.445492 | 1891.853141 | 0.330137 | 0.012374 |
| FURIN | 8290.07203 | 6620.76458 | 0.324237 | 0.037569 |
| CCL25 | 156.1758811 | 125.0785126 | 0.320577 | 0.029303 |
| EFCAB14 | 1671.456108 | 1338.40118 | 0.320571 | 0.014902 |
| TMEM238 | 377.7289751 | 302.3981701 | 0.319547 | 0.002083 |
| HHIP | 731.3470213 | 586.3683347 | 0.318491 | 0.016752 |
| AP1M1 | 1178.897782 | 947.7631034 | 0.31509 | 0.010781 |
| GCLM | 564.9741306 | 454.173877 | 0.314704 | 0.023503 |
| GGA2 | 296.1197708 | 238.1427329 | 0.313518 | 0.016502 |
| GATD3A | 1915.225689 | 1543.973711 | 0.311019 | 0.001854 |
| BGN | 3198.356355 | 2583.543746 | 0.308126 | 0.041764 |
| DPH2 | 174.1142403 | 140.6164068 | 0.307534 | 0.00569 |
| PUS1 | 721.2307505 | 582.8953323 | 0.306271 | 0.001332 |
| ZNF623 | 143.2500522 | 115.7958467 | 0.305791 | 0.025783 |
| BAG5 | 238.7360807 | 192.854623 | 0.305445 | 0.032431 |
| GEMIN2 | 102.4897095 | 83.08930581 | 0.304313 | 0.047399 |
| JMJD8 | 289.7385236 | 234.6780249 | 0.303926 | 0.029352 |
| PCDH18 | 383.964524 | 310.7372451 | 0.303916 | 0.039395 |
| SLC15A4 | 806.7664662 | 653.4862191 | 0.303697 | 0.018202 |
| MSRA | 3417.713998 | 2772.212065 | 0.302048 | 0.012583 |
| FHOD1 | 442.4215412 | 359.3674955 | 0.300766 | 0.010082 |
| ACADS | 9952.137052 | 8095.279538 | 0.29798 | 0.030097 |
| NGFR | 2272.070561 | 1857.182439 | 0.291299 | 0.03685 |
| MXD4 | 827.738619 | 676.8472806 | 0.290762 | 0.012774 |
| LOC507443 | 501.9765682 | 410.8392124 | 0.290746 | 0.023645 |
| NDST1 | 3675.672397 | 3005.69925 | 0.290126 | 0.03761 |
| CRKL | 1727.240195 | 1412.056457 | 0.289833 | 0.003208 |
| MIIP | 739.1404226 | 605.208864 | 0.287795 | 0.002346 |
| IGHMBP2 | 663.5138314 | 544.7405417 | 0.285384 | 0.041245 |
| ND5 | 63580.55225 | 52224.29108 | 0.283863 | 0.025021 |
| ND4L | 15636.97765 | 12859.94961 | 0.282047 | 0.008561 |
| GCH1 | 1203.367639 | 989.5390032 | 0.281648 | 0.04179 |
| UMPS | 462.1459486 | 380.2697285 | 0.280969 | 0.016936 |
| ISOC1 | 1004.190112 | 826.5697032 | 0.280049 | 0.032834 |
| RELN | 1079.326653 | 889.3013245 | 0.279648 | 0.001236 |
| PLSCR3 | 156.9227301 | 129.8296528 | 0.276396 | 0.043647 |
| CYTB | 160410.2516 | 132571.8245 | 0.274993 | 0.044449 |
| ZNF746 | 243.4787672 | 201.1526045 | 0.274203 | 0.040791 |
| ACSM1 | 25198.30949 | 20850.68755 | 0.27324 | 0.019123 |
| SLC22A5 | 269.3744547 | 222.8511826 | 0.272964 | 0.048767 |
| FAM219A | 190.7138293 | 158.0613666 | 0.268906 | 0.04188 |
| DGKQ | 1585.358451 | 1315.494794 | 0.268847 | 0.01858 |
| PM20D1 | 2379.260406 | 1979.004542 | 0.26587 | 0.030804 |
| MDM4 | 1006.636094 | 837.946072 | 0.264826 | 0.033237 |
| RIPK1 | 806.5632092 | 671.2091995 | 0.264529 | 0.019334 |
| BRD1 | 580.9262409 | 483.269296 | 0.262958 | 0.007569 |
| TRAF2 | 848.1575116 | 708.0530658 | 0.261088 | 0.022521 |
| FAM83H | 567.1079242 | 473.9164933 | 0.258035 | 0.033815 |
| KPNA2 | 296.1617011 | 247.9052853 | 0.255986 | 0.04227 |
| RNASET2 | 1006.489197 | 843.8034353 | 0.255326 | 0.019259 |
| NSMCE3 | 554.3615484 | 463.8477688 | 0.255105 | 0.013458 |
| RPUSD4 | 212.175262 | 178.0751915 | 0.253021 | 0.02512 |
| RNPEPL1 | 2214.127559 | 1860.415671 | 0.251529 | 0.045174 |
| HMGCL | 1919.663022 | 1617.510548 | 0.247474 | 0.023192 |
| SLC27A4 | 4828.682301 | 4070.083562 | 0.246487 | 0.018421 |
| SNX15 | 222.842016 | 187.9427804 | 0.246111 | 0.027266 |
| ADI1 | 14088.9146 | 11884.36187 | 0.245558 | 0.045014 |
| VPS37A | 446.1405321 | 376.0072481 | 0.244945 | 0.044218 |
| MTO1 | 134.5117665 | 113.6475324 | 0.241151 | 0.044084 |
| NAXD | 2168.835651 | 1835.259622 | 0.24112 | 0.012704 |
| COQ9 | 935.5429361 | 790.9217071 | 0.241031 | 0.017911 |
| MLYCD | 1473.990909 | 1248.305427 | 0.239928 | 0.007836 |
| TIGD5 | 363.5691807 | 308.4000719 | 0.237345 | 0.03975 |
| GFER | 384.9017161 | 326.7637497 | 0.235318 | 0.033495 |
| NOA1 | 370.1195622 | 314.4210635 | 0.234462 | 0.044323 |
| NADK | 2726.11133 | 2319.093886 | 0.233346 | 0.035127 |
| RBM15B | 424.4355651 | 361.7904792 | 0.229784 | 0.019894 |
| ALDH2 | 33766.68595 | 28804.98931 | 0.229294 | 0.037416 |
| FBRSL1 | 917.3734905 | 782.4000324 | 0.228702 | 0.035488 |
| SLC22A18 | 2116.79207 | 1809.980001 | 0.225977 | 0.008692 |
| TCF7L2 | 390.5738127 | 334.2729598 | 0.22593 | 0.044394 |
| MBD6 | 663.0733424 | 567.8967749 | 0.222244 | 0.040274 |
| IPO13 | 976.2794418 | 836.5098905 | 0.221529 | 0.0103 |
| TSC2 | 949.7468681 | 815.8663341 | 0.218004 | 0.010675 |
| SPIN1 | 643.31384 | 552.6950726 | 0.217658 | 0.031615 |
| ANKRD28 | 321.9991404 | 276.7805918 | 0.217144 | 0.032051 |
| BPHL | 1482.208942 | 1274.886001 | 0.21694 | 0.039947 |
| PMPCA | 3088.199317 | 2661.585032 | 0.214418 | 0.041726 |
| NEK7 | 330.1831612 | 284.3828993 | 0.214348 | 0.0473 |
| TIMM8A | 799.4623464 | 689.3472867 | 0.212545 | 0.041135 |
| RPS6KA4 | 395.4501903 | 341.6120956 | 0.210975 | 0.042059 |
| MVD | 777.60731 | 672.3525798 | 0.2104 | 0.045299 |
| SMNDC1 | 316.3257092 | 273.8653661 | 0.207626 | 0.042984 |
| ATG4B | 414.6110886 | 358.6401734 | 0.207305 | 0.038056 |
| PTGES2 | 831.0362691 | 720.1670705 | 0.206603 | 0.045468 |
| TCN2 | 9090.685711 | 7880.017962 | 0.206271 | 0.009274 |
| MAF1 | 1036.982352 | 901.0011925 | 0.203653 | 0.030247 |
| SLC48A1 | 684.5312423 | 594.6625935 | 0.203651 | 0.048198 |
| BSG | 11464.50776 | 9958.444859 | 0.203162 | 0.045208 |
| ND4 | 121964.1781 | 105956.3323 | 0.202992 | 0.029235 |
| SPNS1 | 918.8705187 | 798.3643393 | 0.202554 | 0.046131 |
| MED15 | 849.2253982 | 742.6450383 | 0.19373 | 0.042487 |
| HPN | 11259.32986 | 9928.609062 | 0.181458 | 0.035936 |
| MIGA2 | 571.7099392 | 505.6219374 | 0.176307 | 0.022348 |
| FBRS | 846.2249949 | 754.4733717 | 0.165095 | 0.049267 |
| SENP2 | 996.2403313 | 1110.323036 | -0.15589 | 0.042971 |
| PRDX5 | 2798.108883 | 3126.671315 | -0.1598 | 0.033108 |
| GPR89A | 541.7882233 | 609.2257588 | -0.16912 | 0.048989 |
| MSH3 | 444.9011704 | 499.7278756 | -0.16917 | 0.046317 |
| PIP4K2B | 641.9076995 | 728.1091047 | -0.18132 | 0.035382 |
| CPSF3 | 335.3156317 | 380.7051057 | -0.18432 | 0.042905 |
| TUBGCP6 | 348.8140981 | 398.6496448 | -0.19259 | 0.044514 |
| PFDN1 | 513.0322376 | 588.6126024 | -0.19792 | 0.047145 |
| RBM25 | 969.8766815 | 1116.660598 | -0.20344 | 0.038817 |
| MPHOSPH8 | 296.950631 | 343.3529811 | -0.20924 | 0.018351 |
| ZMAT2 | 608.2257762 | 703.8773568 | -0.21118 | 0.016243 |
| KARS | 1730.13235 | 2004.515214 | -0.21223 | 0.042986 |
| RBM6 | 505.255158 | 585.8377791 | -0.21356 | 0.033447 |
| COPS9 | 703.7078372 | 817.99069 | -0.21632 | 0.038893 |
| HPX | 294254.9318 | 341910.9259 | -0.21656 | 0.027722 |
| RUFY1 | 239.0921066 | 280.9184275 | -0.23177 | 0.019297 |
| RSF1 | 262.7247266 | 309.38443 | -0.23267 | 0.029048 |
| LSM3 | 328.386068 | 387.8825096 | -0.23868 | 0.035436 |
| ANP32A | 1348.709509 | 1599.335892 | -0.24546 | 0.029323 |
| RBM27 | 225.5660315 | 268.5512976 | -0.2517 | 0.022585 |
| POLB | 138.9262799 | 165.5661708 | -0.25297 | 0.02417 |
| C15H11orf74 | 138.3008214 | 164.9451218 | -0.25519 | 0.036387 |
| IPO8 | 372.0387978 | 445.0436163 | -0.25845 | 0.011935 |
| RPL22 | 2361.721956 | 2825.735351 | -0.25848 | 0.043592 |
| XRN1 | 203.9901121 | 244.6239401 | -0.26088 | 0.032555 |
| TMUB1 | 278.025368 | 335.9816118 | -0.27343 | 0.039757 |
| SMARCA2 | 989.6596638 | 1198.635871 | -0.27586 | 0.026658 |
| MTTP | 2636.201972 | 3196.971069 | -0.27825 | 0.036492 |
| NUDCD1 | 243.4488087 | 295.0996076 | -0.27995 | 0.03127 |
| LOC101906178 | 220.1027815 | 269.4941296 | -0.2883 | 0.016217 |
| ARNT | 120.2184925 | 146.481203 | -0.28868 | 0.011892 |
| KDM1B | 760.4523501 | 929.1742998 | -0.28935 | 0.042156 |
| ANAPC13 | 260.4741266 | 318.7930163 | -0.29261 | 0.005036 |
| COL6A5 | 167.9314018 | 206.3810412 | -0.29924 | 0.019025 |
| C14H8orf59 | 210.9942511 | 259.8592908 | -0.30077 | 0.012021 |
| C3H1orf123 | 253.3934885 | 311.6343769 | -0.30082 | 0.018496 |
| SPCS2 | 4264.950806 | 5258.13458 | -0.30218 | 0.043977 |
| PRDM4 | 140.4705706 | 174.1339435 | -0.30745 | 0.014389 |
| PSMG1 | 382.9638949 | 475.3720512 | -0.31116 | 0.030988 |
| RPA3 | 374.6331153 | 464.8172579 | -0.31178 | 0.022485 |
| ZNF395 | 410.0044715 | 509.2665867 | -0.31212 | 0.012651 |
| SNRPE | 177.4286603 | 220.3793887 | -0.31314 | 0.037439 |
| ARL3 | 129.0461482 | 160.9898946 | -0.31611 | 0.014919 |
| DTD1 | 225.052461 | 281.0607412 | -0.31898 | 0.019728 |
| WDR25 | 73.67416128 | 91.7920977 | -0.31943 | 0.030568 |
| COMMD1 | 170.4764059 | 213.6990552 | -0.32266 | 0.035298 |
| CCDC15 | 181.1131683 | 227.0320668 | -0.32494 | 0.027494 |
| RAP1GDS1 | 180.0334885 | 225.5169149 | -0.32577 | 0.013779 |
| SLX4IP | 132.5166152 | 166.296458 | -0.32737 | 0.037735 |
| PPP1R7 | 296.3649513 | 372.8923259 | -0.32995 | 0.000106 |
| MEF2C | 109.260976 | 138.8203226 | -0.34368 | 0.045455 |
| NCOA7 | 110.6691216 | 140.8242395 | -0.34672 | 0.013867 |
| OSBPL3 | 115.7569049 | 147.3852924 | -0.34778 | 0.022204 |
| TMED8 | 110.0630812 | 140.1779739 | -0.34987 | 0.045949 |
| NAV1 | 104.5715639 | 134.2562265 | -0.35658 | 0.012442 |
| ARHGEF40 | 82.03698522 | 105.7009721 | -0.363 | 0.019122 |
| LOC781381 | 127.4431908 | 164.5992798 | -0.36892 | 0.021767 |
| CDC7 | 117.8148806 | 152.2009048 | -0.37063 | 0.013639 |
| POPDC3 | 543.1665569 | 703.2191198 | -0.37165 | 0.003196 |
| PPP1R16B | 70.13915043 | 91.05533957 | -0.37463 | 0.02851 |
| STARD9 | 48.85646273 | 63.41396015 | -0.37545 | 0.044102 |
| PRRG4 | 312.7460633 | 406.3350586 | -0.37653 | 0.021454 |
| DGKH | 141.394936 | 183.9471884 | -0.37825 | 0.042736 |
| LOC100848538 | 310.0674722 | 404.4259656 | -0.3831 | 0.025422 |
| AHNAK | 1638.363728 | 2138.168988 | -0.38364 | 0.002361 |
| CD14 | 3194.057381 | 4176.325484 | -0.38689 | 0.035155 |
| LOC101905894 | 111.2632038 | 145.6196453 | -0.38876 | 0.044726 |
| MXD1 | 219.7031559 | 287.6303448 | -0.38935 | 0.009958 |
| AFAP1L1 | 76.27165537 | 100.1936739 | -0.39059 | 0.023293 |
| LOC505918 | 151.9347234 | 200.2193773 | -0.3991 | 0.005571 |
| ACVR1 | 305.641455 | 404.5309584 | -0.4056 | 0.009791 |
| GLCCI1 | 180.1265928 | 239.0623075 | -0.4105 | 0.037526 |
| DCDC2 | 213.0515631 | 284.1842631 | -0.41535 | 0.025155 |
| LOC781254 | 124.4049072 | 166.9793276 | -0.42374 | 0.007695 |
| MFHAS1 | 50.78296761 | 68.30859103 | -0.42796 | 0.029948 |
| LOC101903038 | 244.1994379 | 329.0020299 | -0.42913 | 0.022497 |
| RIPOR2 | 50.62241042 | 68.67961354 | -0.44129 | 0.021374 |
| NDUFA12 | 81.74202021 | 111.214015 | -0.44239 | 0.00316 |
| ZNF286A | 255.2008698 | 346.7534426 | -0.44315 | 0.019274 |
| CDK5 | 102.3813249 | 139.4140042 | -0.44466 | 0.002468 |
| LOC104968478 | 77916.31899 | 106069.3157 | -0.44501 | 0.023922 |
| SCML1 | 102.509866 | 139.3960879 | -0.44526 | 0.045544 |
| LOC100847118 | 56.23507809 | 76.64231148 | -0.44639 | 0.016335 |
| LOC112444152 | 261.6681341 | 358.1875655 | -0.45189 | 0.042697 |
| PRDM10 | 62.64100092 | 85.97596538 | -0.45271 | 0.016842 |
| PSTPIP1 | 52.16329364 | 71.93232481 | -0.45742 | 0.029132 |
| ATXN7L1 | 116.7711448 | 160.9797217 | -0.45922 | 0.007421 |
| LCLAT1 | 180.165547 | 247.8191538 | -0.45934 | 0.009539 |
| PKNOX2 | 88.93797098 | 124.4492315 | -0.48059 | 0.016545 |
| FAM160B1 | 45.54710698 | 63.23139641 | -0.48286 | 0.04404 |
| S100A14 | 518.8792418 | 726.9160907 | -0.48567 | 0.00429 |
| ARHGAP23 | 45.12298857 | 63.39709344 | -0.4889 | 0.045708 |
| VWA5A | 194.597818 | 273.2642359 | -0.48994 | 0.023696 |
| DGAT2 | 665.2372309 | 939.2072538 | -0.49723 | 0.005766 |
| ITGA4 | 33.13795225 | 46.705468 | -0.49921 | 0.040544 |
| SLC50A1 | 335.7705025 | 474.8918321 | -0.50078 | 0.01309 |
| LOC100298868 | 1610.58939 | 2280.708271 | -0.50194 | 0.032085 |
| CBFA2T3 | 59.96071192 | 85.06549621 | -0.50251 | 0.028606 |
| LOC100196901 | 87.68453367 | 124.4507405 | -0.5037 | 0.048128 |
| CLIP2 | 36.29507312 | 51.55793551 | -0.50466 | 0.041974 |
| LOC101908015 | 120.078845 | 172.1497225 | -0.51647 | 0.044039 |
| SLC2A3 | 61.56694677 | 88.59713843 | -0.52221 | 0.040372 |
| PCP4L1 | 70.67627138 | 101.529405 | -0.5245 | 0.02012 |
| PCDHGC3 | 75.01262523 | 108.7079217 | -0.5358 | 0.016619 |
| FAM212A | 33.5396119 | 48.88864492 | -0.53585 | 0.046819 |
| ECSCR | 37.61762054 | 54.51056441 | -0.53724 | 0.04737 |
| FHL3 | 25.72064188 | 37.36132546 | -0.53892 | 0.036021 |
| ADH4 | 7537.806321 | 10965.33631 | -0.5407 | 0.036347 |
| CCDC142 | 32.37316312 | 47.31618297 | -0.5437 | 0.025102 |
| KPNA5 | 56.20029687 | 82.05867491 | -0.54876 | 0.005498 |
| LOC783202 | 25.60262446 | 37.65742064 | -0.55053 | 0.049764 |
| LOC616893 | 148.4989767 | 218.6833697 | -0.55588 | 0.004241 |
| LOC112445007 | 21.91117088 | 32.52147141 | -0.55951 | 0.028195 |
| PELI2 | 27.346687 | 40.19080272 | -0.56039 | 0.028698 |
| PLCXD2 | 27.66560503 | 41.03007136 | -0.56393 | 0.029755 |
| GNB4 | 42.62813559 | 63.40192177 | -0.5665 | 0.005983 |
| PHF19 | 44.73892486 | 66.41166591 | -0.57054 | 0.038794 |
| FAM221A | 339.9374224 | 505.4718206 | -0.57341 | 0.02548 |
| LOC784160 | 26.20727578 | 39.58260336 | -0.58743 | 0.022802 |
| LOC101904642 | 26.19700034 | 39.17412251 | -0.58875 | 0.036179 |
| IRAK3 | 173.4456747 | 263.7652031 | -0.60287 | 0.003791 |
| CACNA1H | 146.6805998 | 223.5831551 | -0.60875 | 0.033123 |
| CCSER1 | 140.4061834 | 214.3833622 | -0.60917 | 0.00272 |
| DZIP1 | 57.42802906 | 88.07313834 | -0.61638 | 0.034023 |
| HS3ST1 | 44.59525074 | 68.87371465 | -0.62655 | 0.000901 |
| ITIH3 | 10704.91204 | 16530.28584 | -0.62682 | 0.004221 |
| GAS7 | 23.37250848 | 36.32284102 | -0.63161 | 0.005115 |
| CCDC42 | 200.6280766 | 311.3596212 | -0.6332 | 0.006295 |
| PQLC3 | 25.01910606 | 39.0395357 | -0.63513 | 0.022971 |
| MYOM3 | 57.58465858 | 89.88189646 | -0.64178 | 0.017205 |
| ZBED3 | 82.3824939 | 129.2683279 | -0.64567 | 0.002173 |
| TMED3 | 228.0824695 | 357.9299469 | -0.65053 | 0.022459 |
| ZNF391 | 47.04526548 | 74.93067859 | -0.66495 | 0.008562 |
| IQCG | 21.35147951 | 34.40428662 | -0.67813 | 0.032865 |
| ADGRG6 | 166.0069286 | 267.3042447 | -0.68548 | 0.000468 |
| JCHAIN | 495.2977911 | 803.298693 | -0.69647 | 0.002176 |
| LOC101902226 | 374.2757925 | 608.1488234 | -0.69941 | 0.01743 |
| MECOM | 44.05386374 | 71.78240848 | -0.70154 | 0.003614 |
| MEX3B | 14.2876157 | 23.52993488 | -0.70483 | 0.048132 |
| SPOCK2 | 82.87316677 | 135.7436983 | -0.71031 | 0.017766 |
| ANPEP | 173.4793154 | 284.5455421 | -0.71364 | 0.009606 |
| GLIPR2 | 29.98879586 | 49.50998522 | -0.71957 | 0.043478 |
| COL13A1 | 25.905665 | 42.39693086 | -0.72485 | 0.006787 |
| THBD | 62.27010674 | 102.9549996 | -0.72508 | 0.017995 |
| TMEM156 | 23.29097391 | 38.5274273 | -0.72873 | 0.00704 |
| ACER3 | 43.91462104 | 72.8328273 | -0.73069 | 0.008106 |
| LOC112442664 | 12.20054017 | 20.19131744 | -0.73327 | 0.043973 |
| LOC112448271 | 9.707701187 | 16.10391596 | -0.73433 | 0.039864 |
| ZSCAN26 | 76.29055407 | 127.6963774 | -0.74493 | 0.002104 |
| COL15A1 | 26.92221338 | 45.41475335 | -0.75076 | 0.035469 |
| LOC107133166 | 40.41762739 | 68.18993798 | -0.75628 | 0.007954 |
| LOC107131225 | 9.813406018 | 16.80619964 | -0.76212 | 0.041427 |
| TMEM252 | 9.701585576 | 16.59815653 | -0.76971 | 0.039873 |
| LOC780963 | 40.97701682 | 70.18564604 | -0.77679 | 0.025901 |
| LOC112446757 | 18.32819056 | 31.49085199 | -0.77693 | 0.021741 |
| LOC101906966 | 12.27088619 | 21.17475956 | -0.78642 | 0.02687 |
| SYTL3 | 11.04596516 | 19.30881998 | -0.79571 | 0.022102 |
| LOC787102 | 8.078506764 | 14.24372547 | -0.81472 | 0.039228 |
| VWF | 211.7163352 | 375.5669097 | -0.82653 | 0.003174 |
| ABCG1 | 24.84392758 | 44.25167582 | -0.82771 | 0.021106 |
| DCX | 15.86824142 | 27.95869409 | -0.82844 | 0.006892 |
| LOC112445731 | 12.70980626 | 22.45695781 | -0.82968 | 0.016021 |
| LOC101906281 | 50.44539226 | 89.94681011 | -0.83137 | 0.037282 |
| LOC104975826 | 9.428880213 | 16.87067553 | -0.83612 | 0.04893 |
| RGL3 | 11.49093432 | 20.48760417 | -0.838 | 0.00895 |
| SLC24A3 | 11.91229901 | 21.21042073 | -0.84322 | 0.037939 |
| SMAD3 | 25.29524255 | 46.51590412 | -0.87162 | 0.002792 |
| CEP152 | 9.745130117 | 17.98952347 | -0.87558 | 0.030111 |
| LOC112447495 | 9.435311781 | 17.68985785 | -0.9076 | 0.009553 |
| LOC101906410 | 10.00491502 | 18.97362068 | -0.91534 | 0.021036 |
| IFI44 | 260.5359335 | 493.3046895 | -0.9211 | 0.01689 |
| FUT7 | 6.175260586 | 11.9537603 | -0.94378 | 0.037701 |
| GALNT3 | 8.462814798 | 16.1757784 | -0.95561 | 0.029931 |
| TMEM44 | 9.951339914 | 19.57368946 | -0.9695 | 0.031452 |
| GPC3 | 4793.868851 | 9389.89231 | -0.96988 | 0.00228 |
| LOC107133049 | 32.16529507 | 62.83421855 | -0.97173 | 0.026969 |
| LOC101906657 | 13.40113735 | 26.68455615 | -0.98092 | 0.013128 |
| CCL2 | 24.78422175 | 49.31220161 | -0.99182 | 0.011203 |
| LOC781799 | 7.768440521 | 15.53357522 | -0.99606 | 0.048613 |
| ATP2A1 | 6.441868989 | 12.85351899 | -1.00584 | 0.0263 |
| CABCOCO1 | 5.194912144 | 10.71691527 | -1.03732 | 0.036232 |
| LOC100300832 | 9.731855748 | 20.18016813 | -1.04349 | 0.017598 |
| NR4A1 | 39.93895465 | 82.67268414 | -1.05107 | 0.010428 |
| ACE | 11.19874029 | 23.31532057 | -1.07044 | 0.022525 |
| APOD | 67.04246287 | 141.5037053 | -1.07797 | 0.010115 |
| LOC100847119 | 261.0457616 | 554.2984409 | -1.08564 | 0.034509 |
| CLEC5A | 6.385312349 | 13.6532068 | -1.09683 | 0.048619 |
| LOC112444965 | 3.402367049 | 7.334584373 | -1.09709 | 0.04368 |
| LOC101902861 | 13.87679783 | 30.22055886 | -1.12319 | 8.92E-05 |
| LOC112441904 | 6.062391619 | 13.11414157 | -1.12688 | 0.041747 |
| FSTL3 | 9.368685973 | 20.60733488 | -1.13451 | 0.02676 |
| LOC101903252 | 7.970492962 | 17.8251722 | -1.14391 | 0.036904 |
| PDZD9 | 5.443172941 | 12.24395215 | -1.15457 | 0.011998 |
| LOC783604 | 4.299697751 | 9.789386945 | -1.18385 | 0.04637 |
| MS4A3 | 7.544461383 | 17.26628173 | -1.19652 | 0.009785 |
| FOS | 253.5051691 | 589.4052703 | -1.21765 | 0.047935 |
| LOC112444775 | 9.892206028 | 23.59339147 | -1.24402 | 0.009404 |
| CCDC114 | 4.527009973 | 10.96036228 | -1.26248 | 0.035274 |
| IQCA1 | 4.487302059 | 10.94326276 | -1.26483 | 0.046604 |
| YJEFN3 | 2.51078088 | 6.247496417 | -1.27292 | 0.047616 |
| LOC112446366 | 2.712774318 | 6.658501754 | -1.27656 | 0.037058 |
| ASB9 | 6.549331898 | 16.03183593 | -1.27684 | 0.042163 |
| LOC112442273 | 5.04551731 | 12.71265035 | -1.31762 | 0.006512 |
| MX1 | 1269.167217 | 3184.44437 | -1.32717 | 0.017975 |
| S1PR4 | 7.044393192 | 18.00876932 | -1.32924 | 0.002526 |
| LOC104975134 | 5.082761736 | 13.03976939 | -1.33998 | 0.018516 |
| HPCAL4 | 21.93642387 | 55.6236523 | -1.34829 | 0.018677 |
| LOC101903030 | 38.18507588 | 97.63223264 | -1.35006 | 0.001833 |
| MSMB | 12.18542336 | 31.07185675 | -1.35144 | 0.007178 |
| LOC614923 | 4.998249957 | 12.89965902 | -1.35832 | 0.040426 |
| LOC112445197 | 3.124138571 | 8.249448123 | -1.37371 | 0.023842 |
| ENTPD2 | 2.942791588 | 7.808752627 | -1.37826 | 0.028133 |
| LOC525649 | 2.106040882 | 5.270744349 | -1.37841 | 0.042391 |
| OMP | 16.20634006 | 42.20335965 | -1.38222 | 0.001097 |
| GPR55 | 4.389032785 | 11.7989831 | -1.40251 | 0.004129 |
| RASGRF2 | 5.461757604 | 14.59614913 | -1.4192 | 0.006031 |
| SLC14A1 | 3.722345828 | 10.29218338 | -1.43564 | 0.008793 |
| EGR1 | 223.3731748 | 606.8490913 | -1.44234 | 0.024208 |
| EDN1 | 6.073625955 | 16.79644768 | -1.45975 | 0.013704 |
| LOC112442248 | 2.262690855 | 6.339828124 | -1.47613 | 0.020035 |
| LOC101907606 | 7.843638709 | 22.04002946 | -1.48986 | 0.024217 |
| CCDC152 | 7.038540388 | 19.85812306 | -1.4928 | 0.018582 |
| ISLR | 3.662870464 | 10.48194951 | -1.50039 | 0.036169 |
| PLAG1 | 6.558451397 | 18.67288805 | -1.51843 | 0.000391 |
| LOC107131239 | 11.15893183 | 32.84535272 | -1.55303 | 0.026604 |
| LOC112441609 | 2.920312786 | 9.148224008 | -1.65403 | 0.01833 |
| RAPSN | 2.190978161 | 7.119038661 | -1.65927 | 0.027548 |
| LOC100336734 | 4.257942979 | 13.65680146 | -1.669 | 0.049849 |
| CSDC2 | 2.538721352 | 8.280869856 | -1.67496 | 0.016998 |
| LOC112449404 | 1.817977892 | 5.907403337 | -1.67736 | 0.021871 |
| LOC107132843 | 1.735750302 | 5.547091413 | -1.68556 | 0.040712 |
| FOSB | 13.9692141 | 45.28099388 | -1.70155 | 0.012888 |
| LOC101904916 | 1.767691653 | 5.897301324 | -1.75603 | 0.04295 |
| CDH20 | 2.320298205 | 7.990741003 | -1.7874 | 0.013747 |
| LOC101904902 | 1.598761169 | 5.416443996 | -1.79938 | 0.022646 |
| LOC104969653 | 1.893871951 | 6.524349293 | -1.80161 | 0.006889 |
| IGF2BP2 | 1.304576351 | 4.426294738 | -1.81389 | 0.044803 |
| LOC522763 | 12.17415947 | 43.01069123 | -1.82257 | 0.005891 |
| PPP1R42 | 1.450273057 | 5.288020101 | -1.82984 | 0.035904 |
| MYCN | 2.584500942 | 9.502895596 | -1.8843 | 0.008407 |
| PRRX1 | 1.750267393 | 6.427490715 | -1.88878 | 0.029263 |
| JAZF1 | 1.412695938 | 5.387283298 | -1.946 | 0.01874 |
| HTR1E | 1.219944515 | 4.782755179 | -1.95669 | 0.031169 |
| SRCIN1 | 1.124004032 | 4.29643111 | -1.95995 | 0.036927 |
| CEP55 | 1.993275782 | 7.812124905 | -1.96239 | 0.012547 |
| CDCA8 | 1.496904733 | 6.243036106 | -2.02191 | 0.013318 |
| LOC112441500 | 2.815460645 | 11.51326931 | -2.04179 | 0.001336 |
| LOC101906923 | 0.92935608 | 3.895851024 | -2.06427 | 0.025526 |
| TENM1 | 2.006673471 | 8.516580028 | -2.08062 | 0.002158 |
| MIR2284U | 1.23713138 | 5.28848241 | -2.0936 | 0.009195 |
| PRPH2 | 0.797437961 | 3.34240544 | -2.09421 | 0.037952 |
| LOC101905400 | 1.371357777 | 5.990673361 | -2.10225 | 0.009735 |
| NTS | 4.008188255 | 17.63245488 | -2.12215 | 0.009486 |
| LOC539693 | 1.886860579 | 8.337092605 | -2.15438 | 0.001065 |
| LOC107131772 | 0.804834916 | 3.545871533 | -2.17071 | 0.040902 |
| CCL1 | 1.215184774 | 5.786225125 | -2.24022 | 0.025676 |
| LOC101903413 | 0.810524378 | 3.888016196 | -2.28246 | 0.047967 |
| KIAA1549 | 0.783165778 | 3.830593245 | -2.30155 | 0.032179 |
| SLITRK6 | 1.530869275 | 7.393379687 | -2.34436 | 0.004778 |
| LOC101907749 | 0.806442581 | 4.163989637 | -2.3937 | 0.028674 |
| LOC107132098 | 0.467311821 | 2.471726039 | -2.40025 | 0.040163 |
| VN2R401P | 0.94756187 | 4.996633181 | -2.40872 | 0.027578 |
| PABPC1L | 0.467263461 | 2.570516676 | -2.45517 | 0.045779 |
| COL4A3 | 1.234953626 | 6.895257921 | -2.48739 | 0.000809 |
| LOC509415 | 0.33012614 | 2.156488234 | -2.68636 | 0.043393 |
| CXHXorf65 | 0.528756059 | 3.081488115 | -2.69424 | 0.022462 |
| LOC112445985 | 0.436734134 | 3.027284405 | -2.70531 | 0.019325 |
| LOC100300624 | 0.438297196 | 2.987853867 | -2.71042 | 0.03526 |
| LOC104974339 | 0.286839332 | 2.288567091 | -2.77825 | 0.028729 |
| LOC112446377 | 0.478265088 | 3.314187446 | -2.8264 | 0.037039 |
| LOC112447727 | 0.148920479 | 1.760491463 | -2.88066 | 0.046806 |
| TSNAXIP1 | 0.29862249 | 2.453891456 | -2.88121 | 0.027407 |
| LOC101903026 | 0.958093238 | 7.084355787 | -2.90643 | 0.011115 |
| LOC101909551 | 1.115973735 | 8.35559993 | -2.91817 | 0.000548 |
| LOC101903309 | 0.555572703 | 4.66344225 | -2.97257 | 0.01127 |
| LOC112448741 | 0.449106031 | 3.670415018 | -2.97874 | 0.020526 |
| PI15 | 1.044453833 | 8.544118983 | -3.00082 | 0.003894 |
| LOC112447764 | 0.277786351 | 2.834070428 | -3.08418 | 0.022244 |
| LOC112447441 | 0.277786351 | 2.909204176 | -3.13347 | 0.020681 |
| TFAP2B | 0.147946156 | 2.210328653 | -3.2179 | 0.038381 |
| LOC101906511 | 0.317609811 | 3.128757887 | -3.23228 | 0.007344 |
| LOC112442589 | 0 | 1.647797738 | -3.28387 | 0.04201 |
| DEFB7 | 0.880379811 | 8.908684493 | -3.31414 | 0.029146 |
| LOC112444795 | 0 | 1.776341724 | -3.3728 | 0.030453 |
| LOC112447770 | 0.31682828 | 3.45545302 | -3.38777 | 0.016419 |
| CA12 | 0.286839332 | 3.547824967 | -3.42452 | 0.004189 |
| RAB9B | 0 | 1.86326985 | -3.45233 | 0.048827 |
| ULBP27 | 0.138893176 | 2.773461678 | -3.53974 | 0.016695 |
| LOC112449614 | 0 | 2.011104408 | -3.54616 | 0.044887 |
| LOC112443231 | 0 | 2.296429974 | -3.74475 | 0.02952 |
| LOC112441536 | 0.468237784 | 6.216884621 | -3.75092 | 0.001621 |
| FMR1NB | 0.14970201 | 3.426158584 | -3.83857 | 0.009759 |
| MEOX1 | 0.147946156 | 3.819184674 | -3.99312 | 0.004456 |
| RBM46 | 0 | 3.504724532 | -4.35175 | 0.021971 |
| LOC101905242 | 0.777476316 | 20.31422573 | -4.71433 | 3.86E-05 |
| CATHL1 | 0 | 11.12248045 | -6.022 | 0.045334 |
| LOC112441458 | 0 | 12.03021043 | -6.13524 | 0.041404 |
| CATHL2 | 0.295892313 | 28.41975534 | -6.57109 | 0.028781 |
